# Supplementary material for: Climate Change and Mental Health: An Interactive Educational Session
Source: MedEdPORTAL. 2024 Apr 19;20:11418. doi: 10.15766/mep_2374-8265.11418 (PMC11026302; doi:10.15766/mep_2374-8265.11418)
Supplement: Supplementary file 1 — Session Presentation.pptxFacilitator Guide.docxPostsession Resources for Students.docxPre- and Postsession Survey.docx [file mep_2374-8265.11418-s001.zip › A. Session Presentation.pptx]

## Slide 1
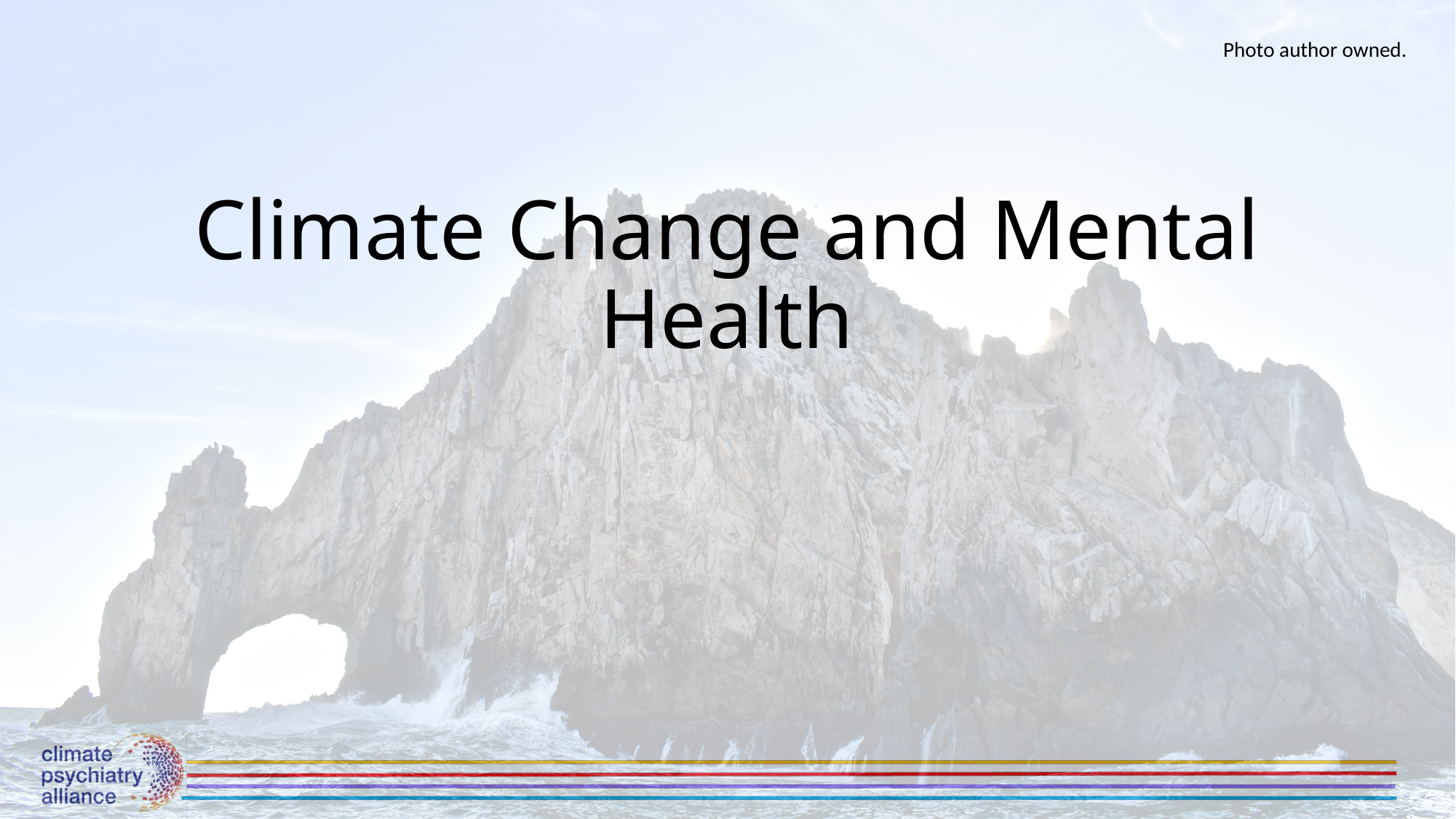

# Climate Change and Mental Health
Photo author owned.

## Slide 2
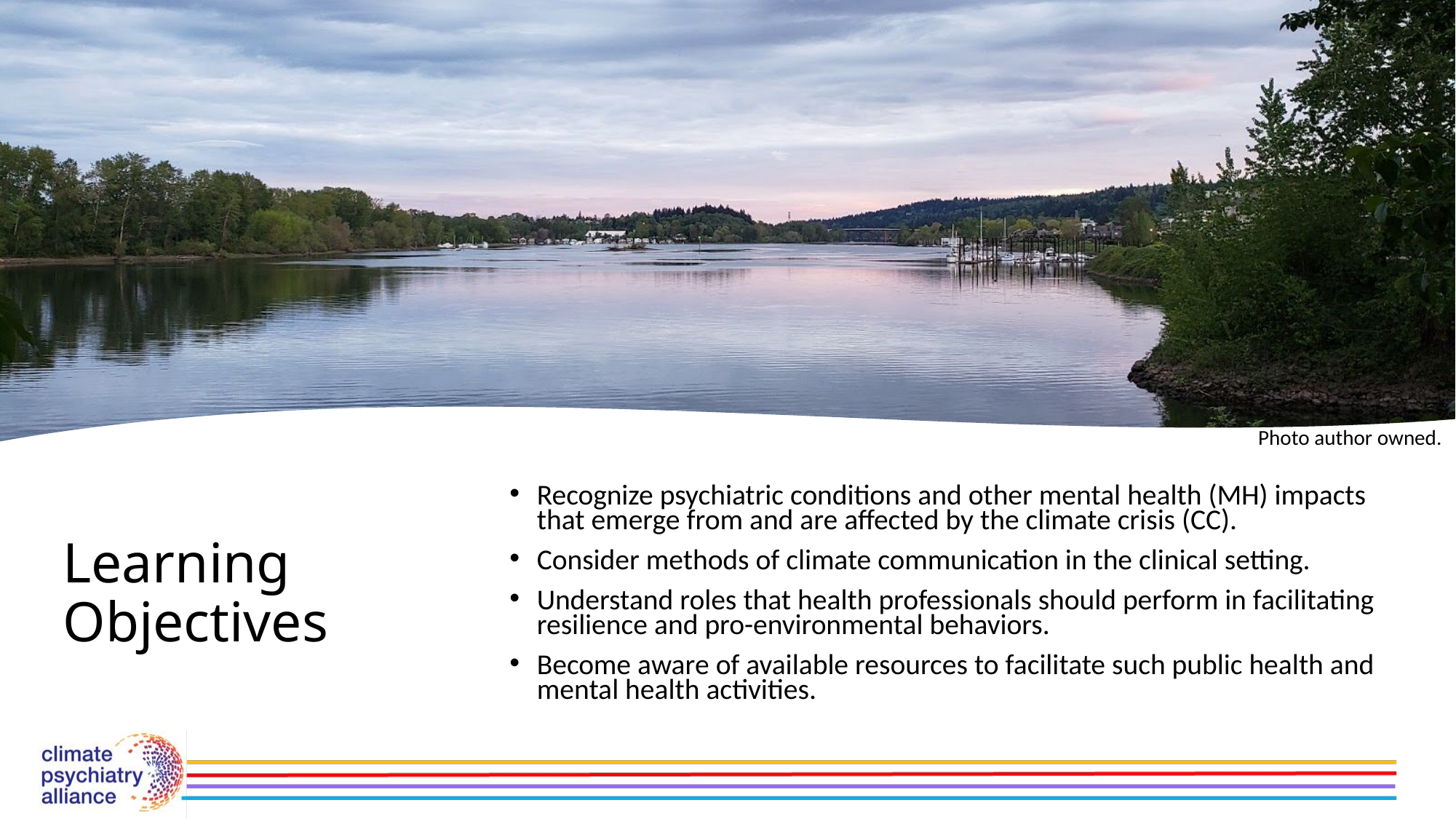

Photo author owned.
# Learning Objectives
Recognize psychiatric conditions and other mental health (MH) impacts that emerge from and are affected by the climate crisis (CC).
Consider methods of climate communication in the clinical setting.
Understand roles that health professionals should perform in facilitating resilience and pro-environmental behaviors.
Become aware of available resources to facilitate such public health and mental health activities.

## Slide 3
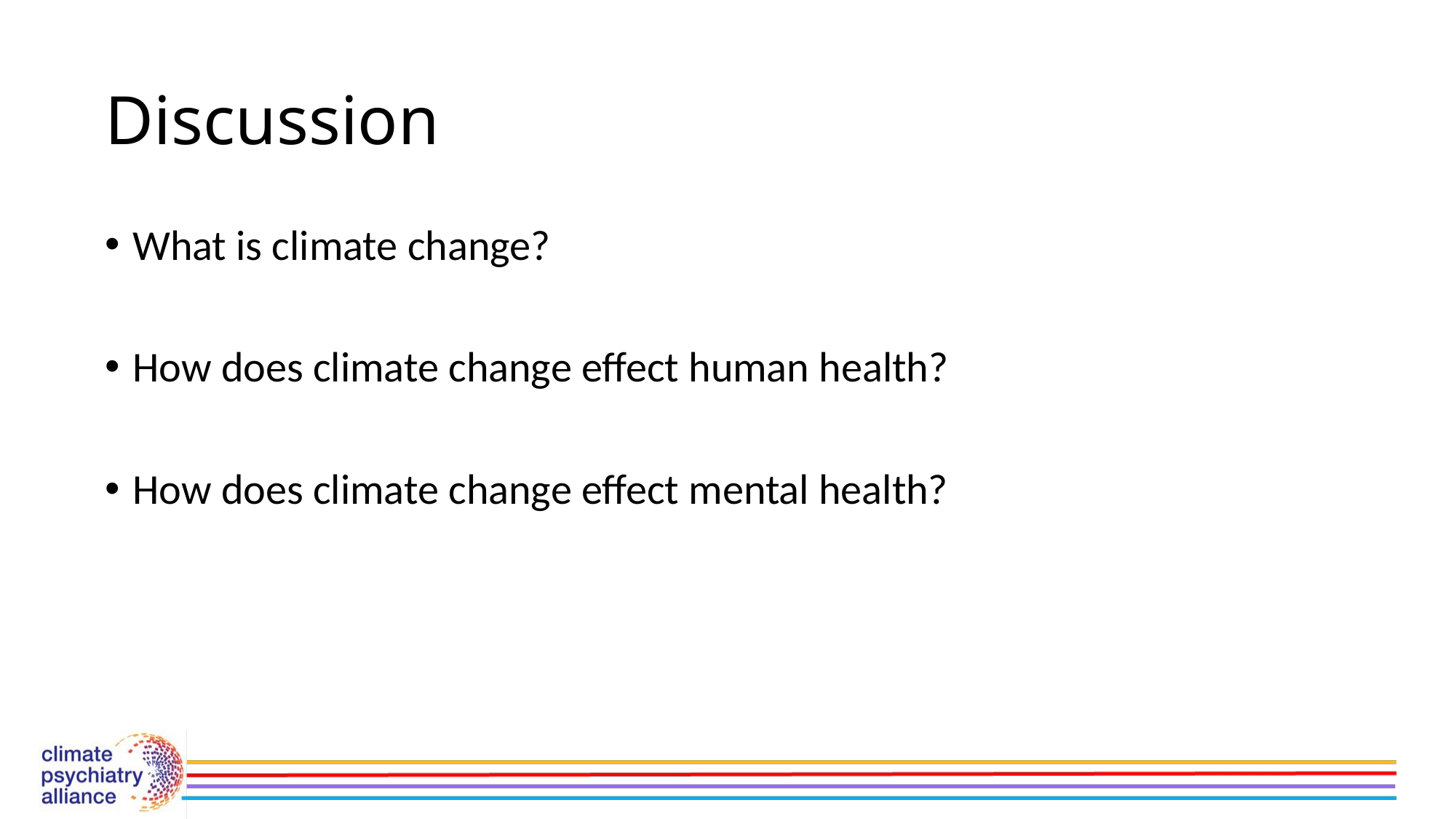

# Discussion
What is climate change?
How does climate change effect human health?
How does climate change effect mental health?

## Slide 4
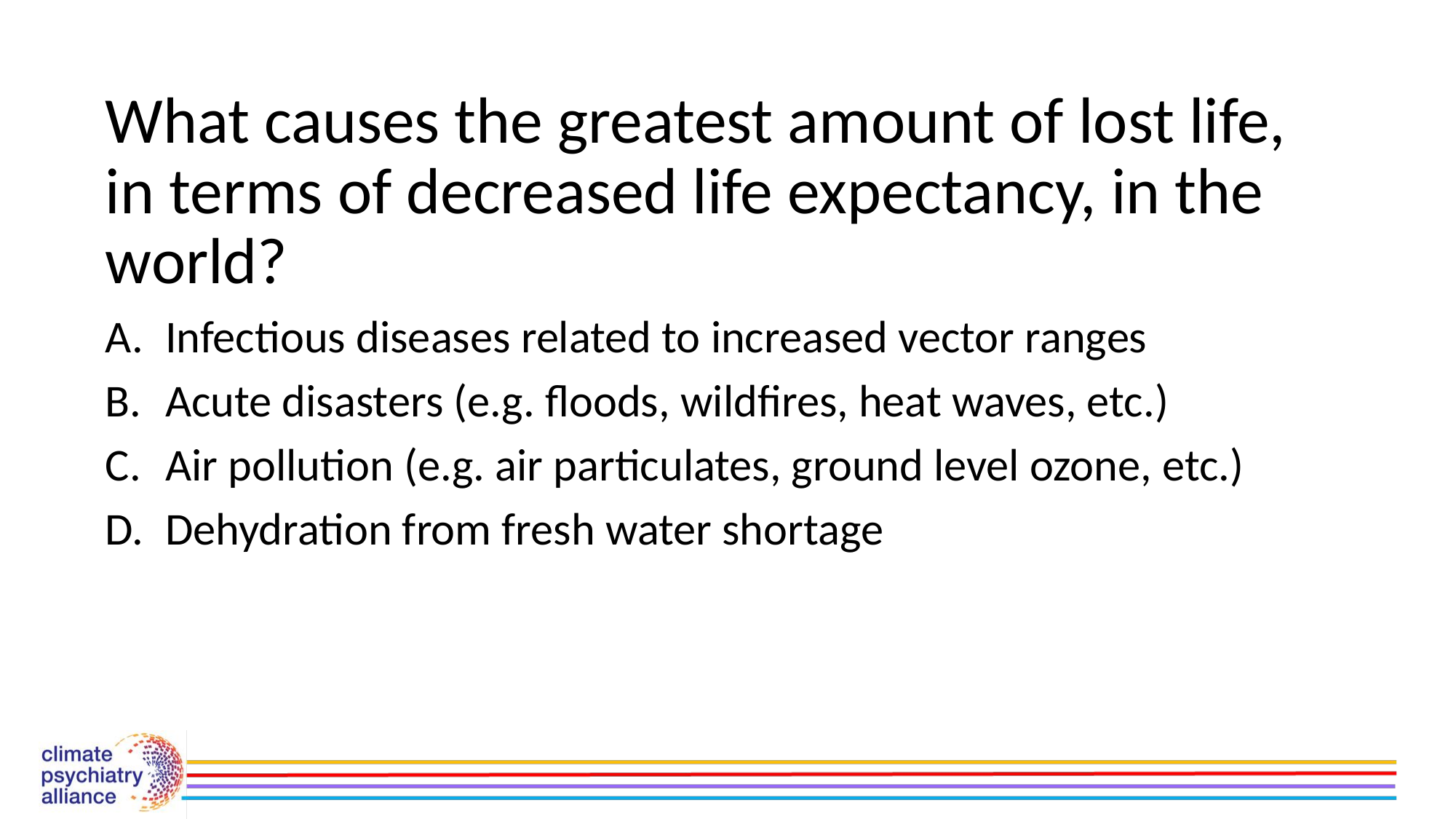

What causes the greatest amount of lost life, in terms of decreased life expectancy, in the world?
Infectious diseases related to increased vector ranges
Acute disasters (e.g. floods, wildfires, heat waves, etc.)
Air pollution (e.g. air particulates, ground level ozone, etc.)
Dehydration from fresh water shortage

## Slide 5
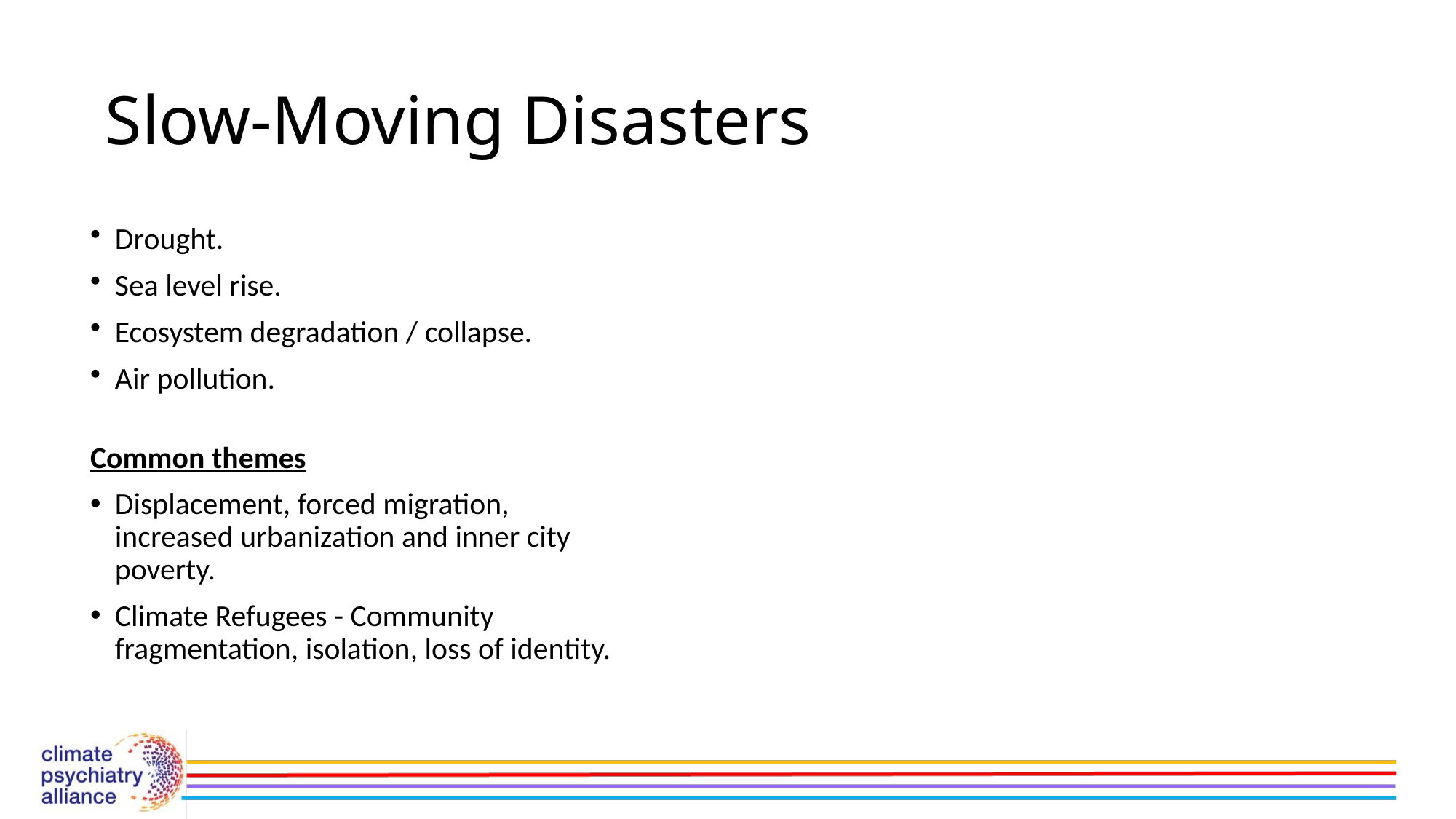

# Slow-Moving Disasters
Drought.
Sea level rise.
Ecosystem degradation / collapse.
Air pollution.
Common themes
Displacement, forced migration, increased urbanization and inner city poverty.
Climate Refugees - Community fragmentation, isolation, loss of identity.

## Slide 6
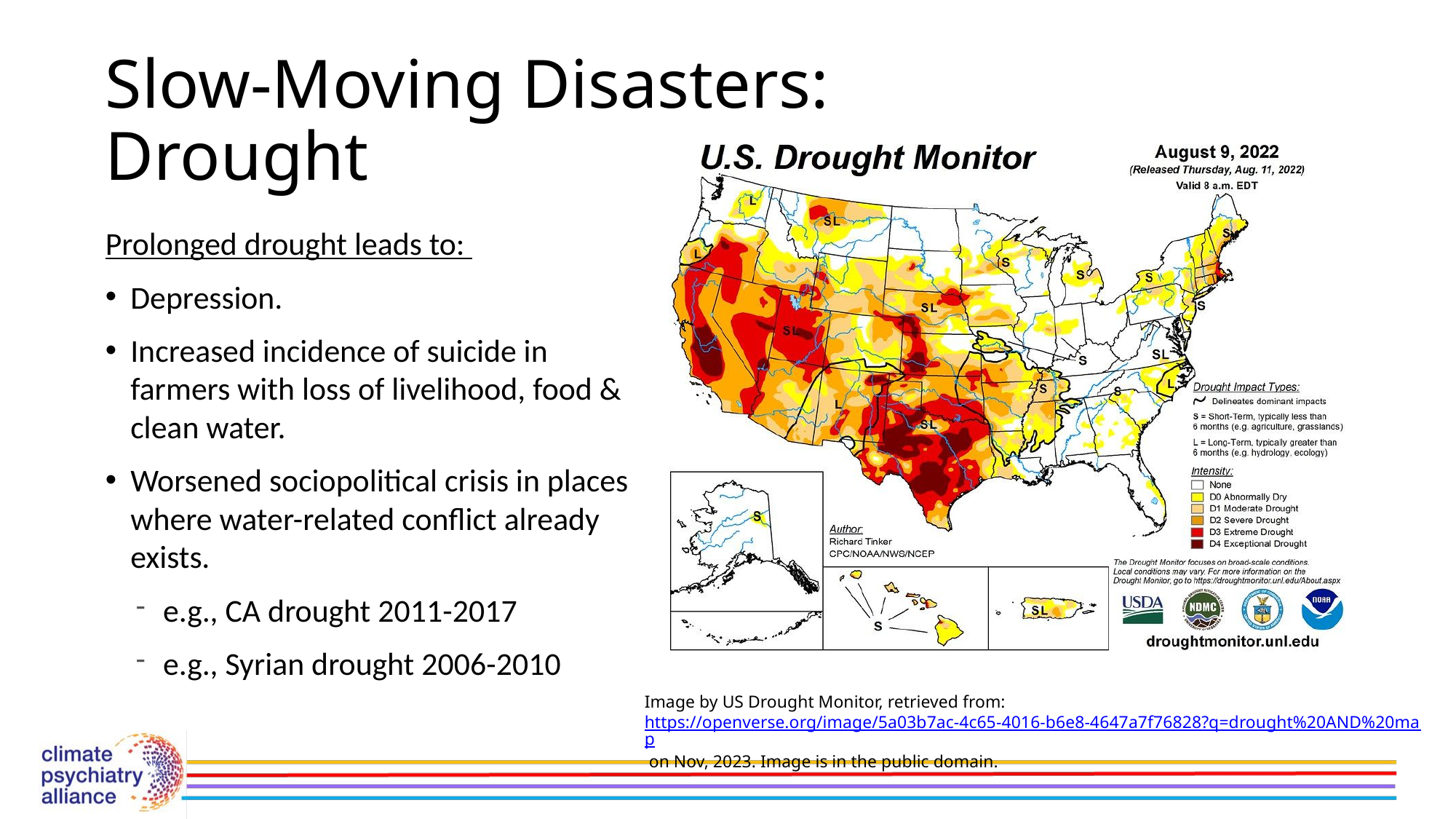

# Slow-Moving Disasters:
Drought
Prolonged drought leads to:
Depression.
Increased incidence of suicide in farmers with loss of livelihood, food & clean water.
Worsened sociopolitical crisis in places where water-related conflict already exists.
e.g., CA drought 2011-2017
e.g., Syrian drought 2006-2010
Image by US Drought Monitor, retrieved from: https://openverse.org/image/5a03b7ac-4c65-4016-b6e8-4647a7f76828?q=drought%20AND%20map on Nov, 2023. Image is in the public domain.

## Slide 7
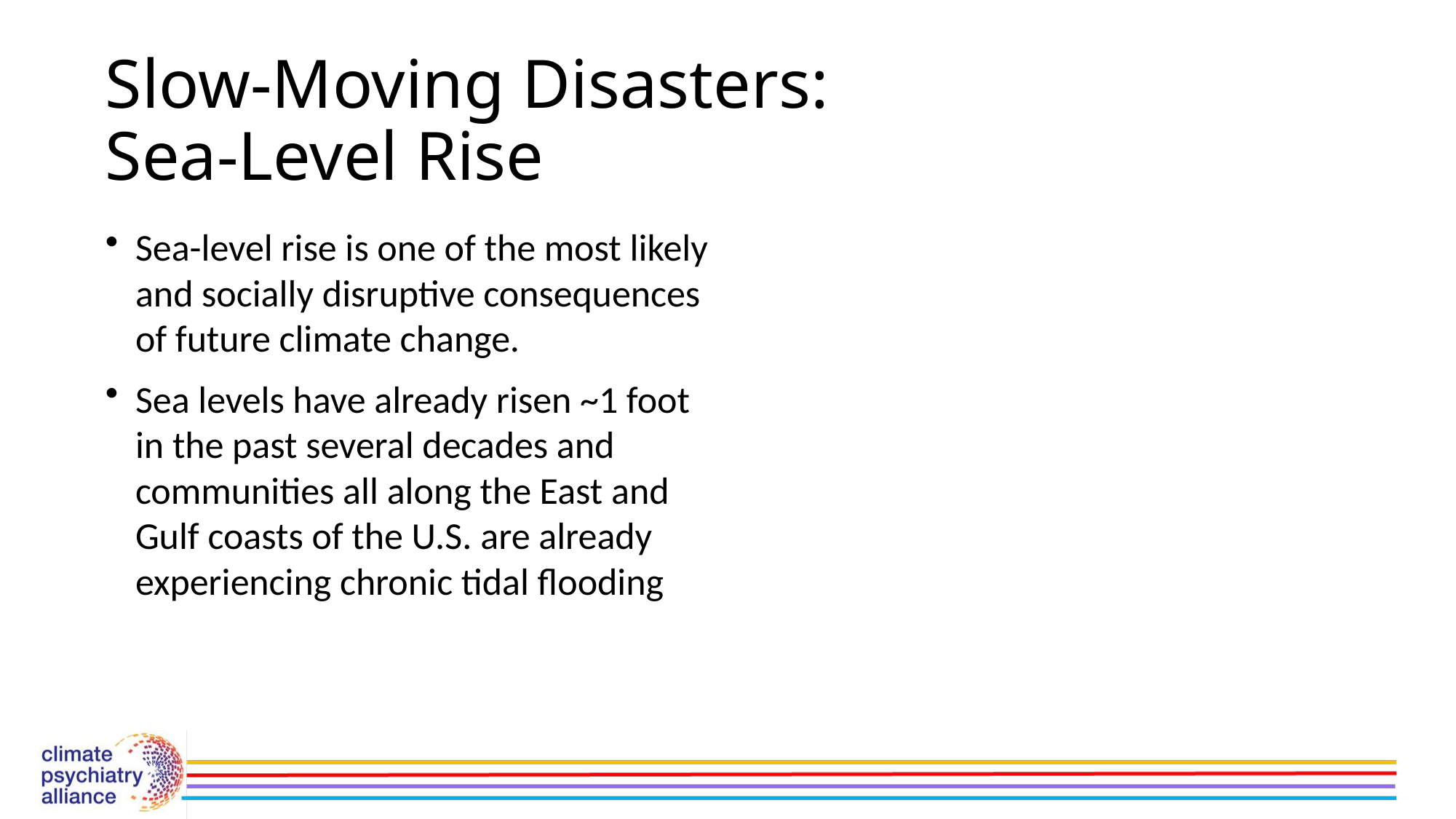

# Slow-Moving Disasters:
Sea-Level Rise
Sea-level rise is one of the most likely and socially disruptive consequences of future climate change.
Sea levels have already risen ~1 foot in the past several decades and communities all along the East and Gulf coasts of the U.S. are already experiencing chronic tidal flooding

## Slide 8
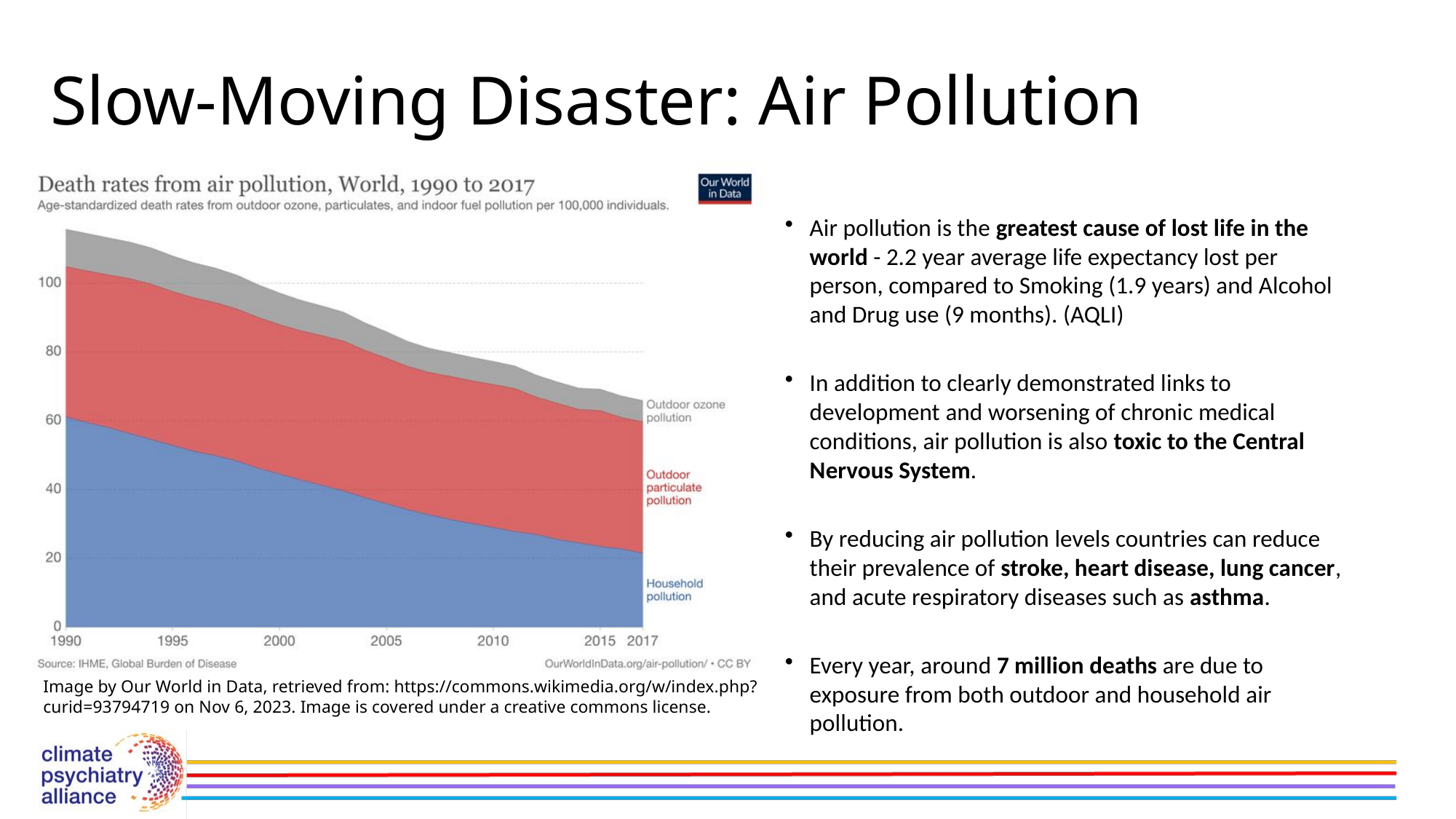

# Slow-Moving Disaster: Air Pollution
Air pollution is the greatest cause of lost life in the world - 2.2 year average life expectancy lost per person, compared to Smoking (1.9 years) and Alcohol and Drug use (9 months). (AQLI)
In addition to clearly demonstrated links to development and worsening of chronic medical conditions, air pollution is also toxic to the Central Nervous System.
By reducing air pollution levels countries can reduce their prevalence of stroke, heart disease, lung cancer, and acute respiratory diseases such as asthma.
Every year, around 7 million deaths are due to exposure from both outdoor and household air pollution.
Image by Our World in Data, retrieved from: https://commons.wikimedia.org/w/index.php?curid=93794719 on Nov 6, 2023. Image is covered under a creative commons license.

## Slide 9
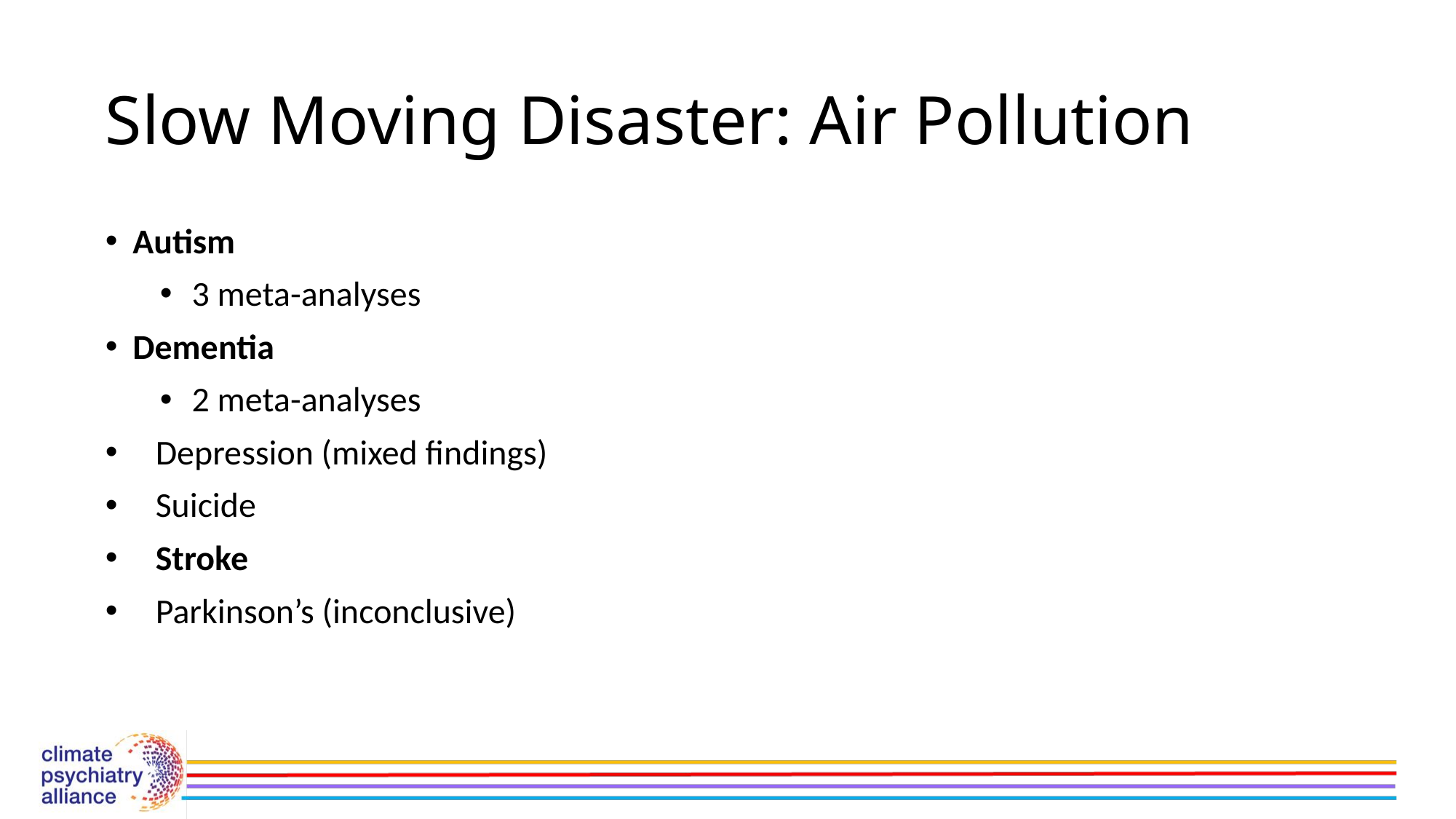

# Slow Moving Disaster: Air Pollution
Autism
3 meta-analyses
Dementia
2 meta-analyses
Depression (mixed findings)
Suicide
Stroke
Parkinson’s (inconclusive)

## Slide 10
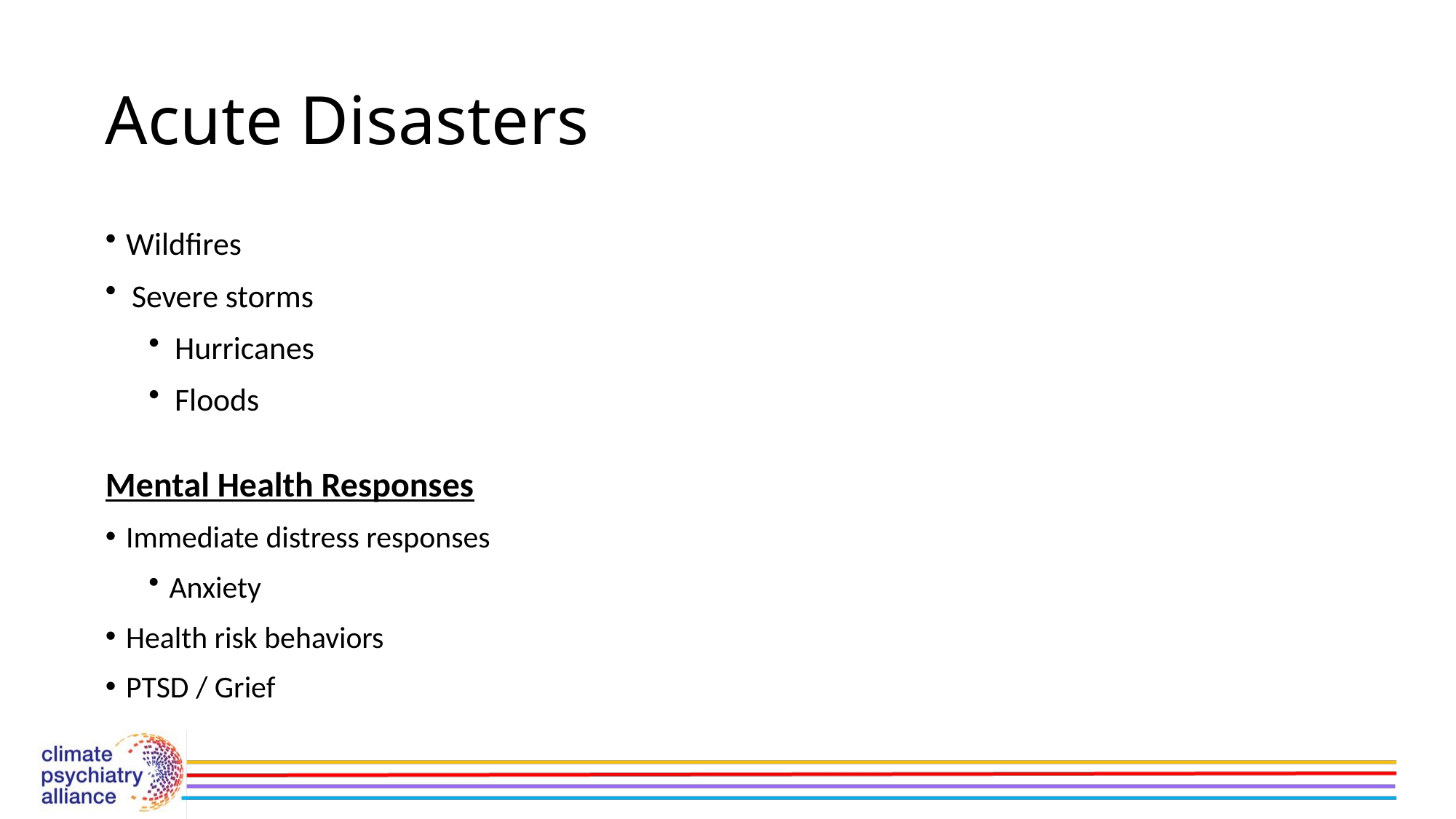

# Acute Disasters
Wildfires
Severe storms
Hurricanes
Floods
Mental Health Responses
Immediate distress responses
Anxiety
Health risk behaviors
PTSD / Grief

## Slide 11
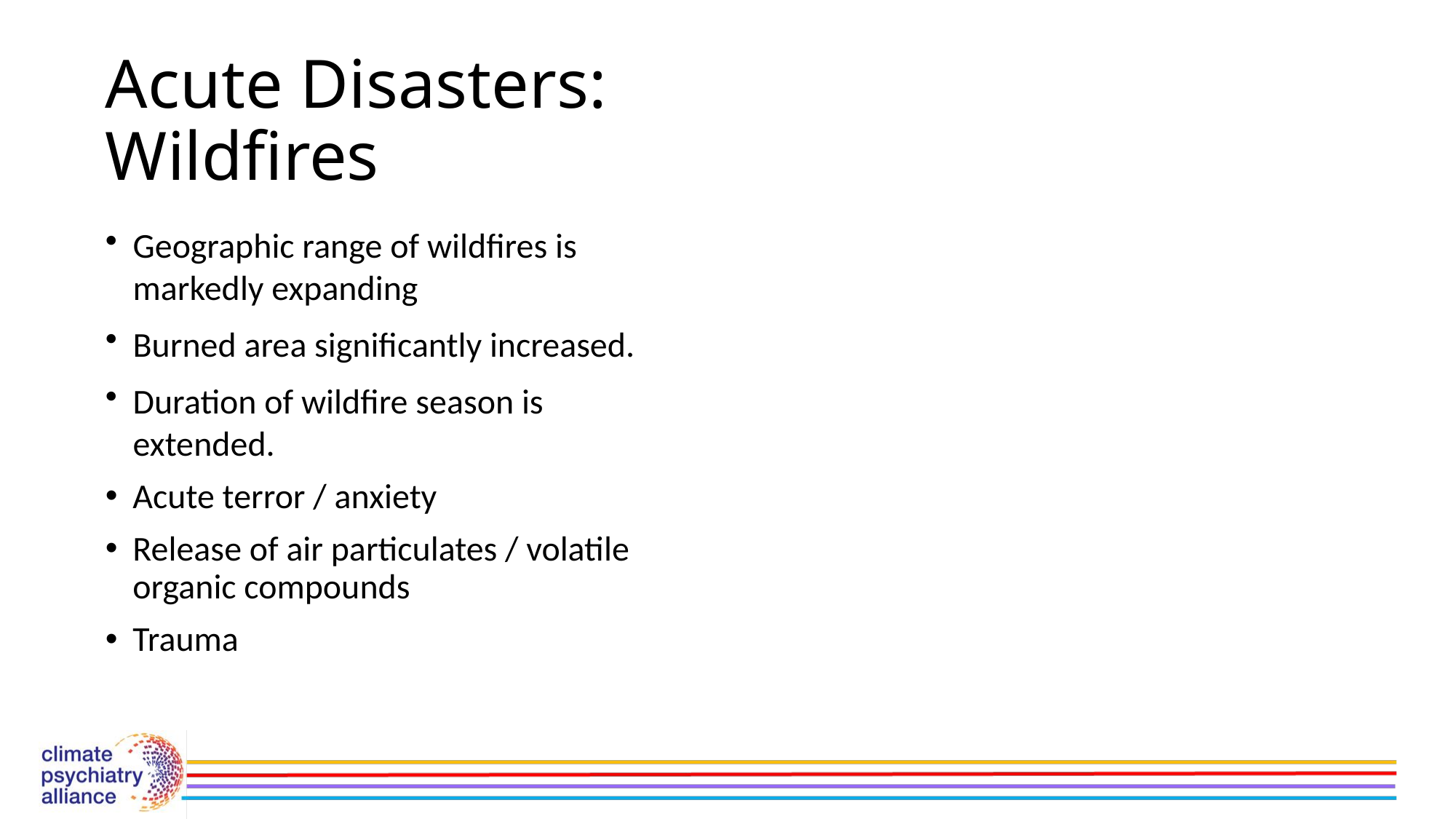

# Acute Disasters:
Wildfires
Geographic range of wildfires is markedly expanding
Burned area significantly increased.
Duration of wildfire season is extended.
Acute terror / anxiety
Release of air particulates / volatile organic compounds
Trauma

## Slide 12
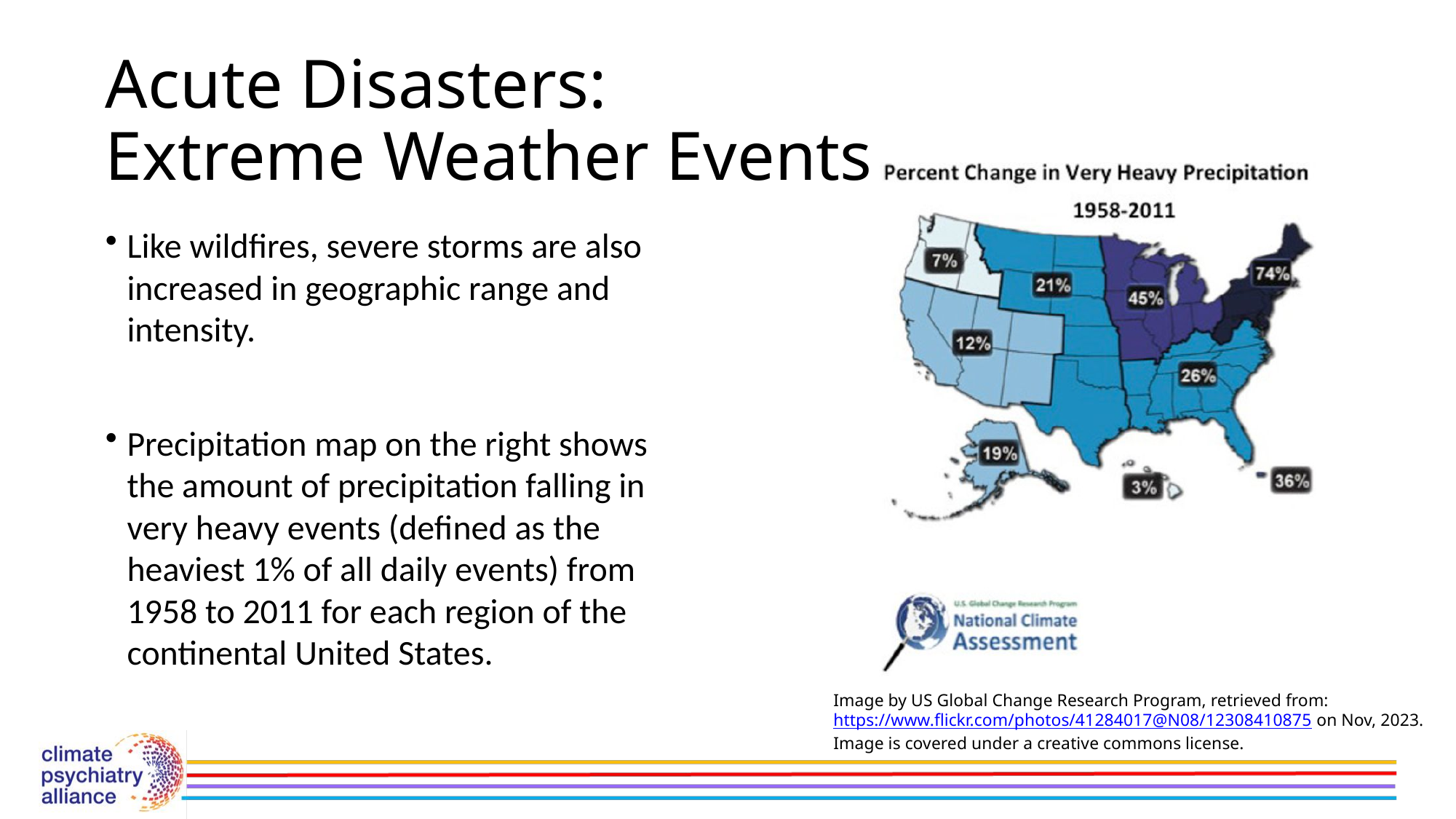

# Acute Disasters:
Extreme Weather Events
Like wildfires, severe storms are also increased in geographic range and intensity.
Precipitation map on the right shows the amount of precipitation falling in very heavy events (defined as the heaviest 1% of all daily events) from 1958 to 2011 for each region of the continental United States.
Image by US Global Change Research Program, retrieved from: https://www.flickr.com/photos/41284017@N08/12308410875 on Nov, 2023. Image is covered under a creative commons license.

## Slide 13
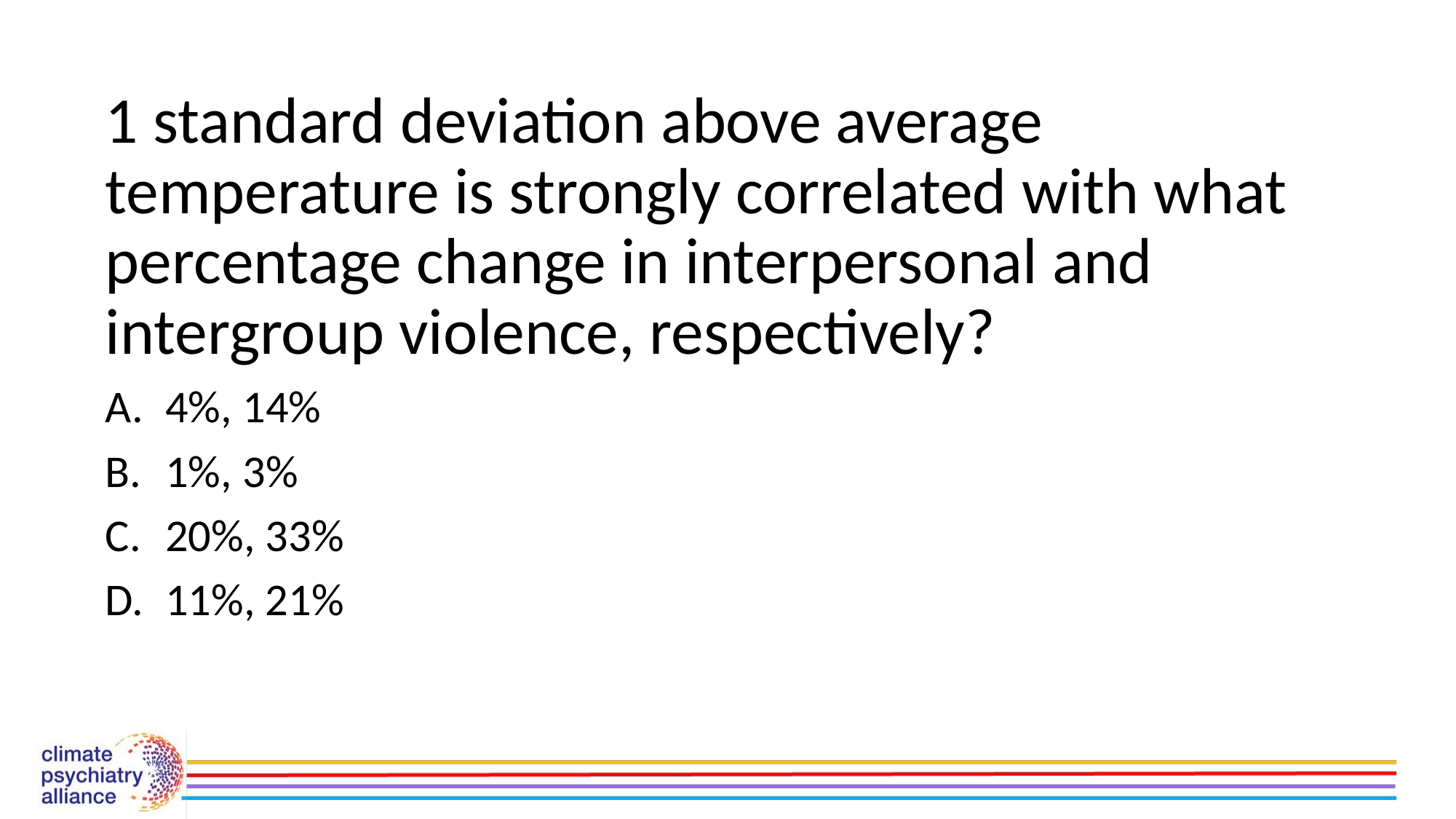

1 standard deviation above average temperature is strongly correlated with what percentage change in interpersonal and intergroup violence, respectively?
4%, 14%
1%, 3%
20%, 33%
11%, 21%

## Slide 14
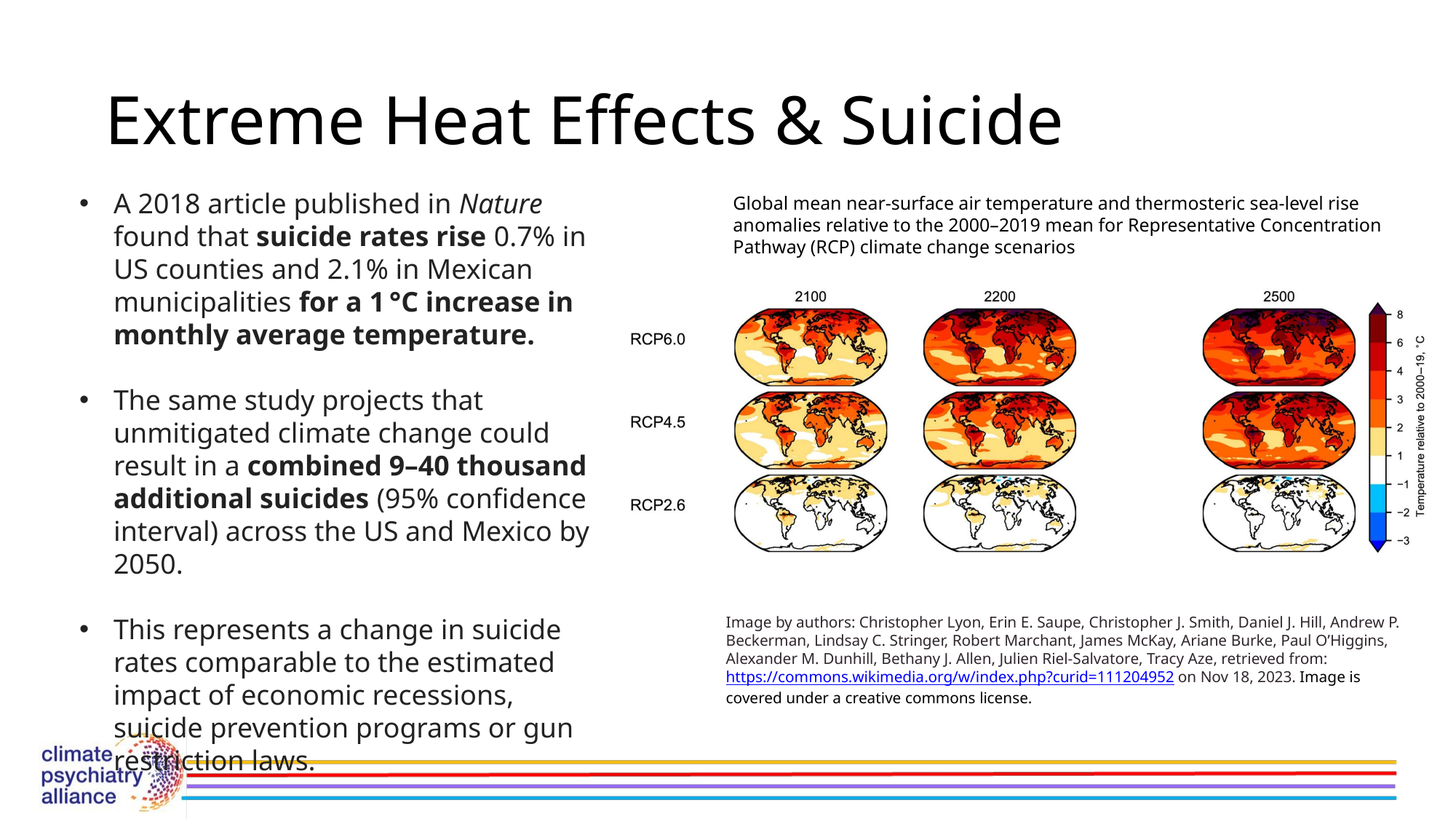

# Extreme Heat Effects & Suicide
A 2018 article published in Nature found that suicide rates rise 0.7% in US counties and 2.1% in Mexican municipalities for a 1 °C increase in monthly average temperature.
The same study projects that unmitigated climate change could result in a combined 9–40 thousand additional suicides (95% confidence interval) across the US and Mexico by 2050.
This represents a change in suicide rates comparable to the estimated impact of economic recessions, suicide prevention programs or gun restriction laws.
Global mean near-surface air temperature and thermosteric sea-level rise anomalies relative to the 2000–2019 mean for Representative Concentration Pathway (RCP) climate change scenarios
Image by authors: Christopher Lyon, Erin E. Saupe, Christopher J. Smith, Daniel J. Hill, Andrew P. Beckerman, Lindsay C. Stringer, Robert Marchant, James McKay, Ariane Burke, Paul O’Higgins, Alexander M. Dunhill, Bethany J. Allen, Julien Riel-Salvatore, Tracy Aze, retrieved from: https://commons.wikimedia.org/w/index.php?curid=111204952 on Nov 18, 2023. Image is covered under a creative commons license.

## Slide 15
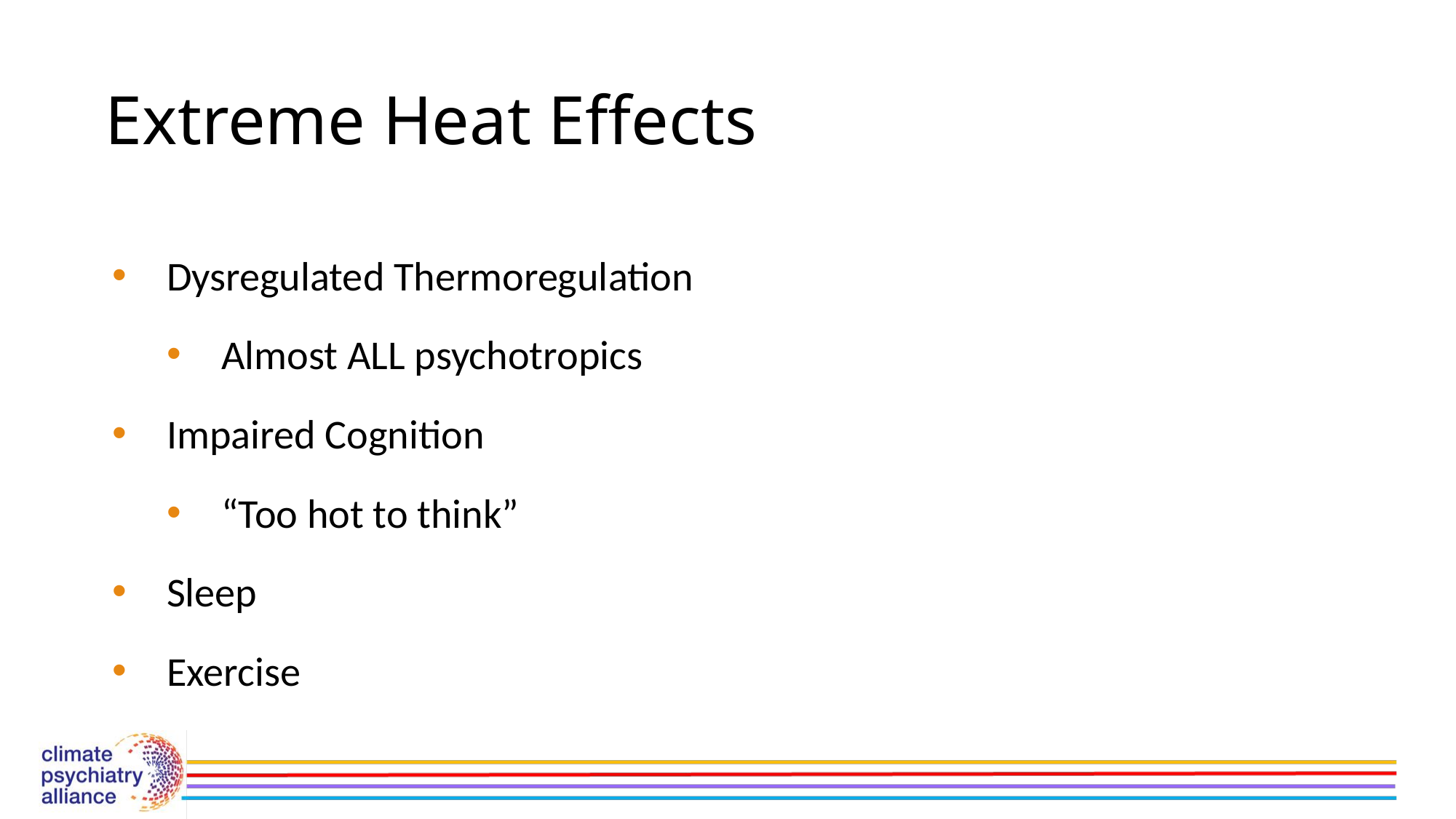

# Extreme Heat Effects
Dysregulated Thermoregulation
Almost ALL psychotropics
Impaired Cognition
“Too hot to think”
Sleep
Exercise

## Slide 16
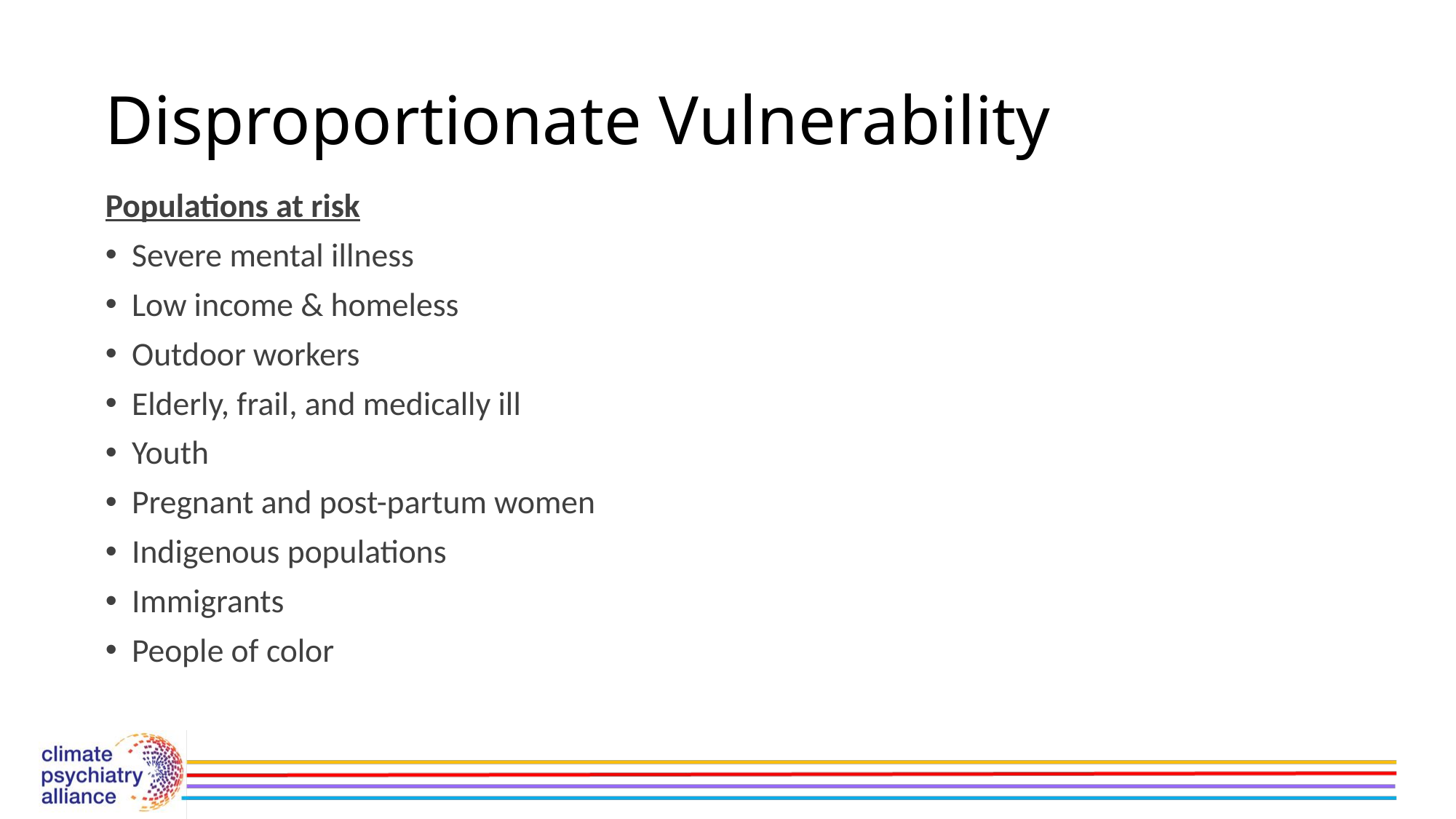

# Disproportionate Vulnerability
Populations at risk
Severe mental illness
Low income & homeless
Outdoor workers
Elderly, frail, and medically ill
Youth
Pregnant and post-partum women
Indigenous populations
Immigrants
People of color

## Slide 17
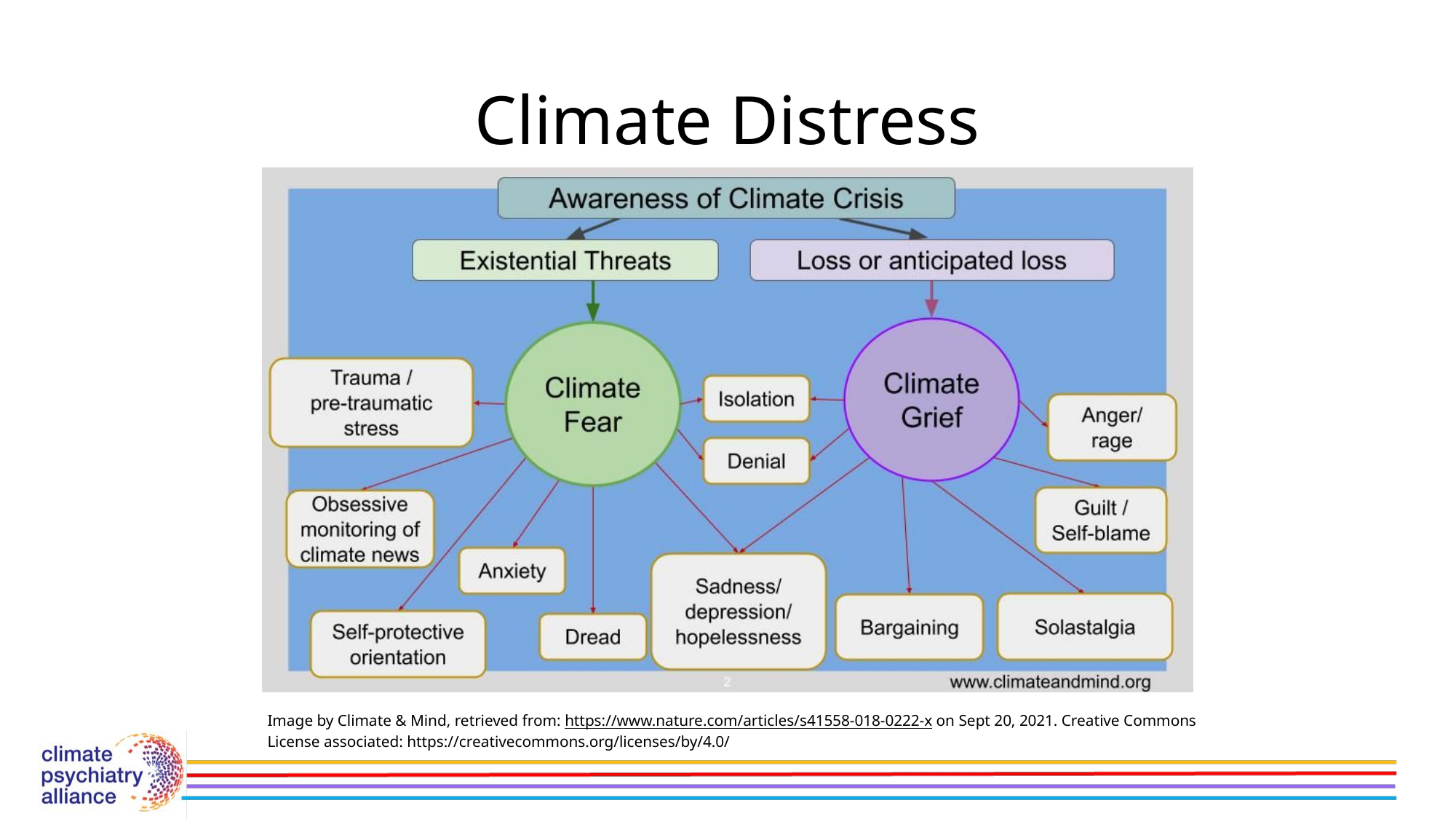

# Climate Distress
Image by Climate & Mind, retrieved from: https://www.nature.com/articles/s41558-018-0222-x on Sept 20, 2021. Creative Commons License associated: https://creativecommons.org/licenses/by/4.0/

## Slide 18
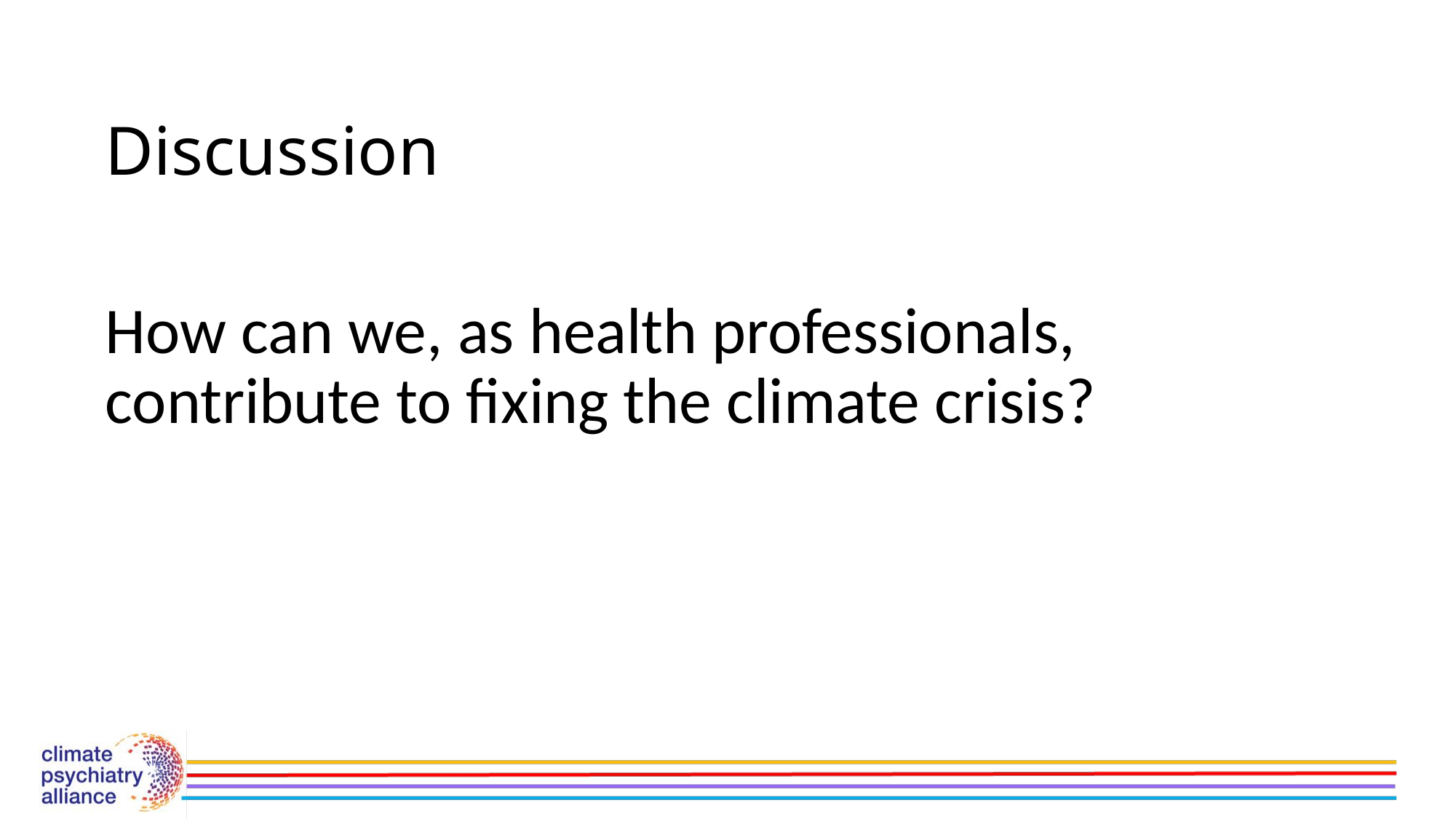

# Discussion
How can we, as health professionals, contribute to fixing the climate crisis?

## Slide 19
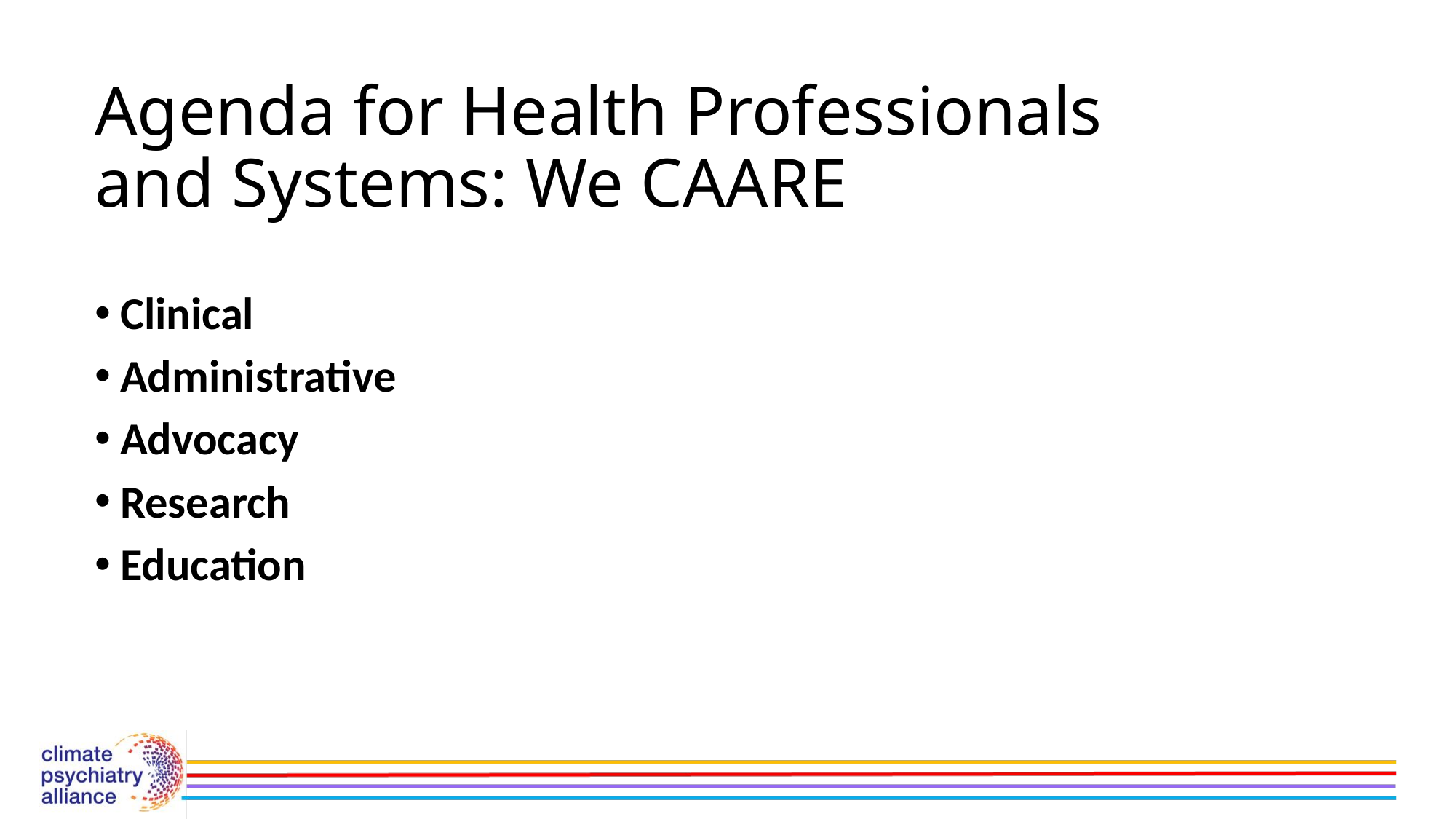

# Agenda for Health Professionals and Systems: We CAARE
Clinical
Administrative
Advocacy
Research
Education

## Slide 20
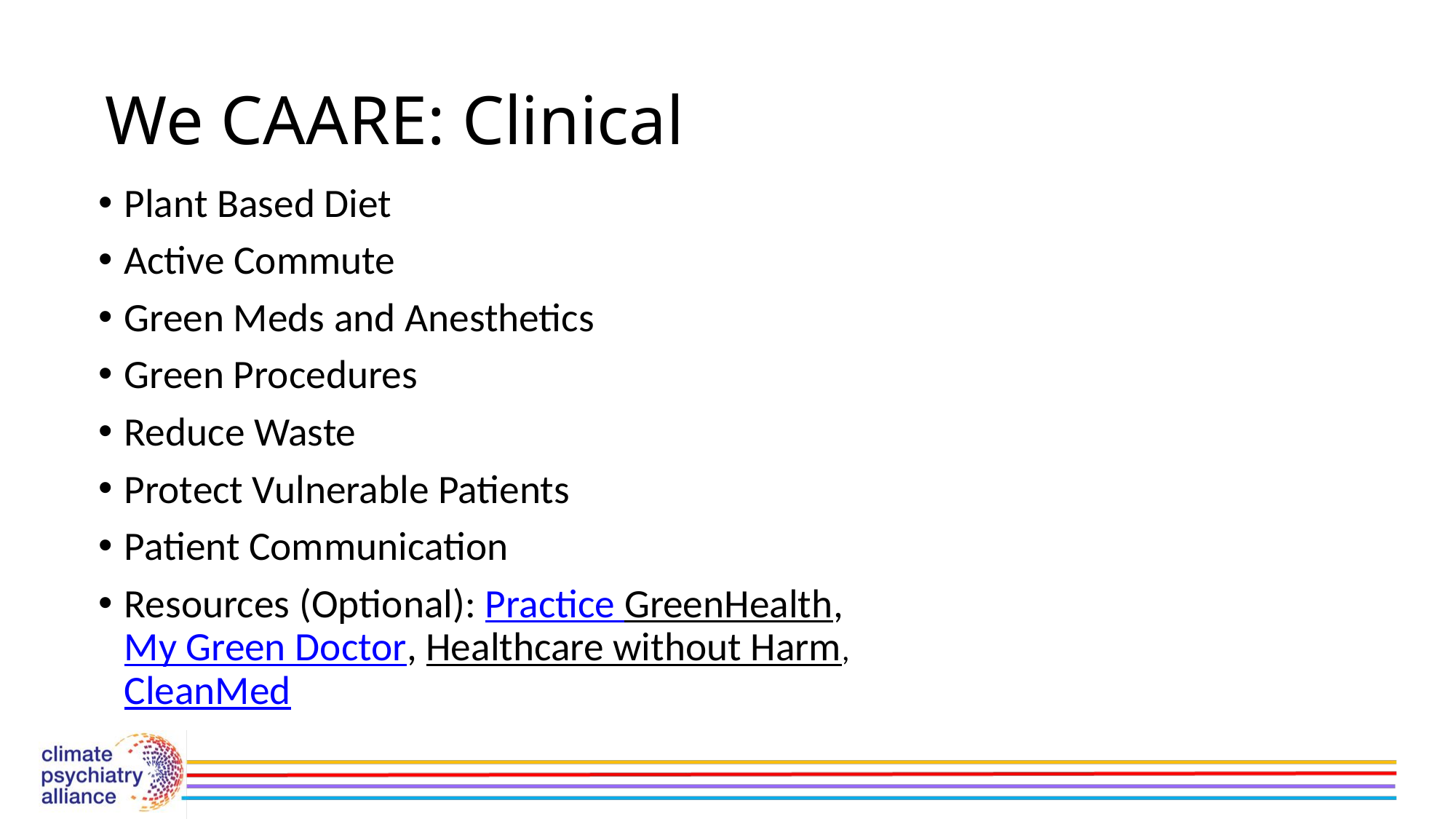

# We CAARE: Clinical
Plant Based Diet
Active Commute
Green Meds and Anesthetics
Green Procedures
Reduce Waste
Protect Vulnerable Patients
Patient Communication
Resources (Optional): Practice GreenHealth, My Green Doctor, Healthcare without Harm, CleanMed

## Slide 21
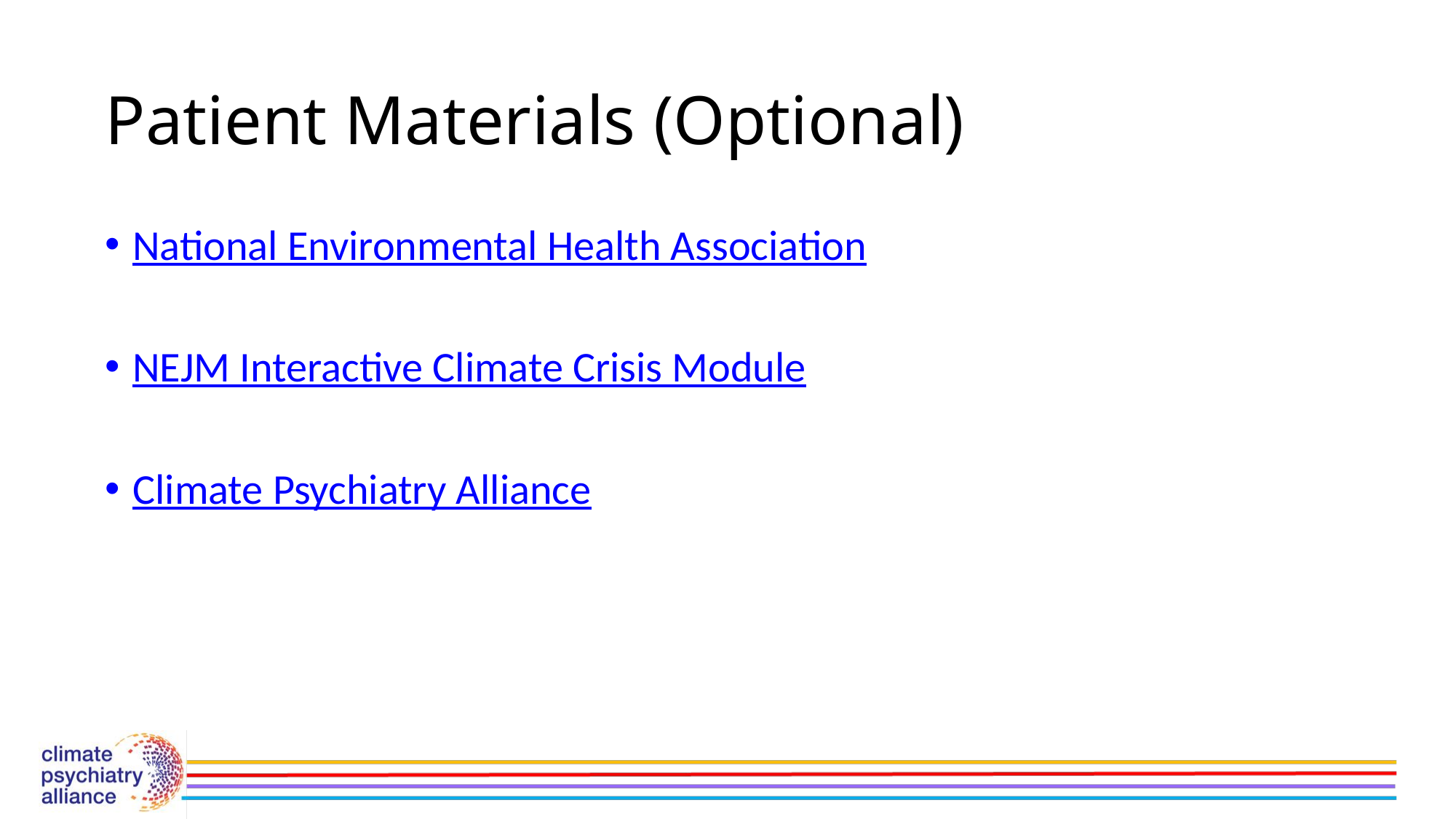

# Patient Materials (Optional)
National Environmental Health Association
NEJM Interactive Climate Crisis Module
Climate Psychiatry Alliance

## Slide 22
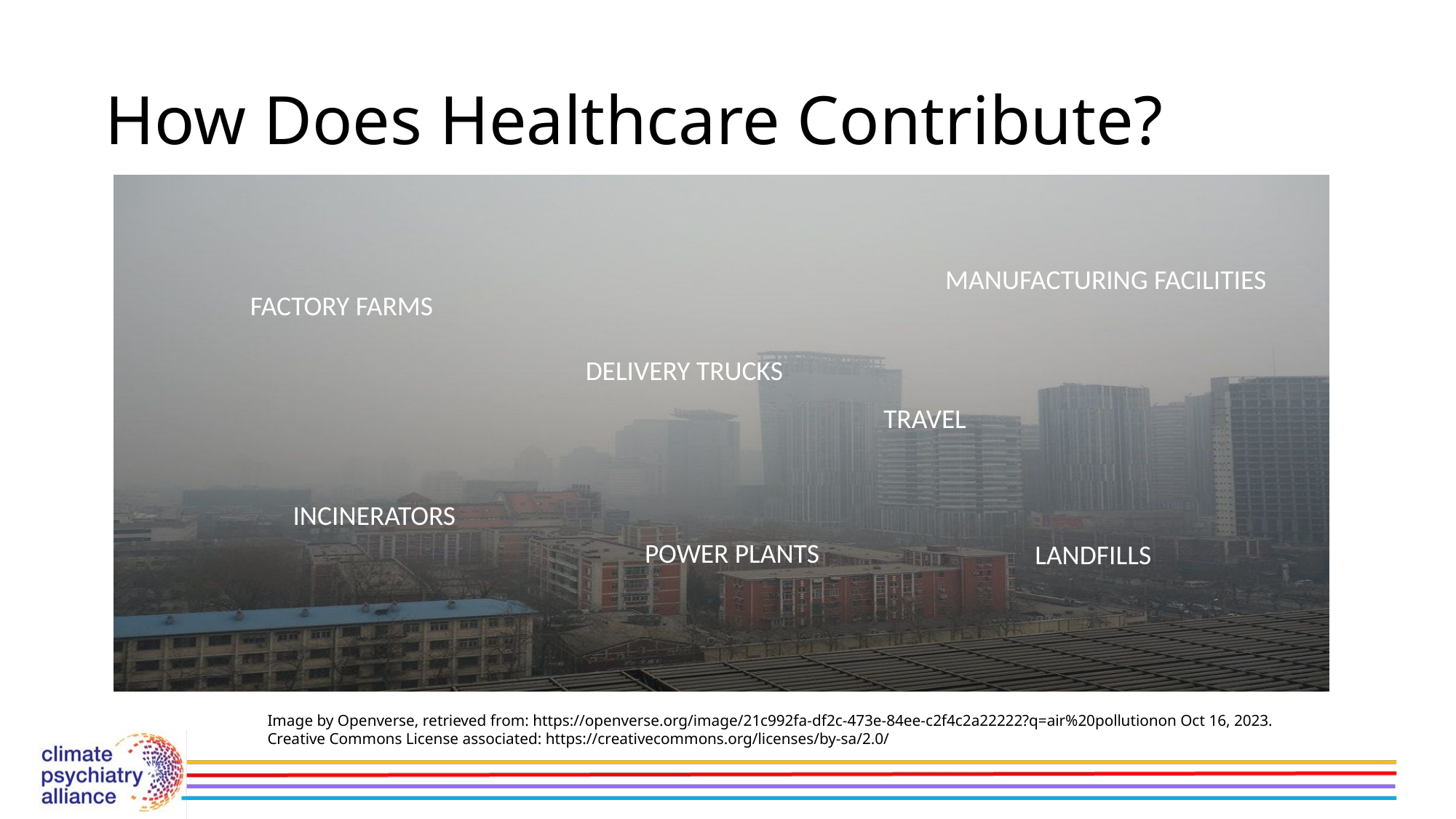

# How Does Healthcare Contribute?
MANUFACTURING FACILITIES
FACTORY FARMS
DELIVERY TRUCKS
TRAVEL
INCINERATORS
POWER PLANTS
LANDFILLS
Image by Openverse, retrieved from: https://openverse.org/image/21c992fa-df2c-473e-84ee-c2f4c2a22222?q=air%20pollutionon Oct 16, 2023. Creative Commons License associated: https://creativecommons.org/licenses/by-sa/2.0/

## Slide 23
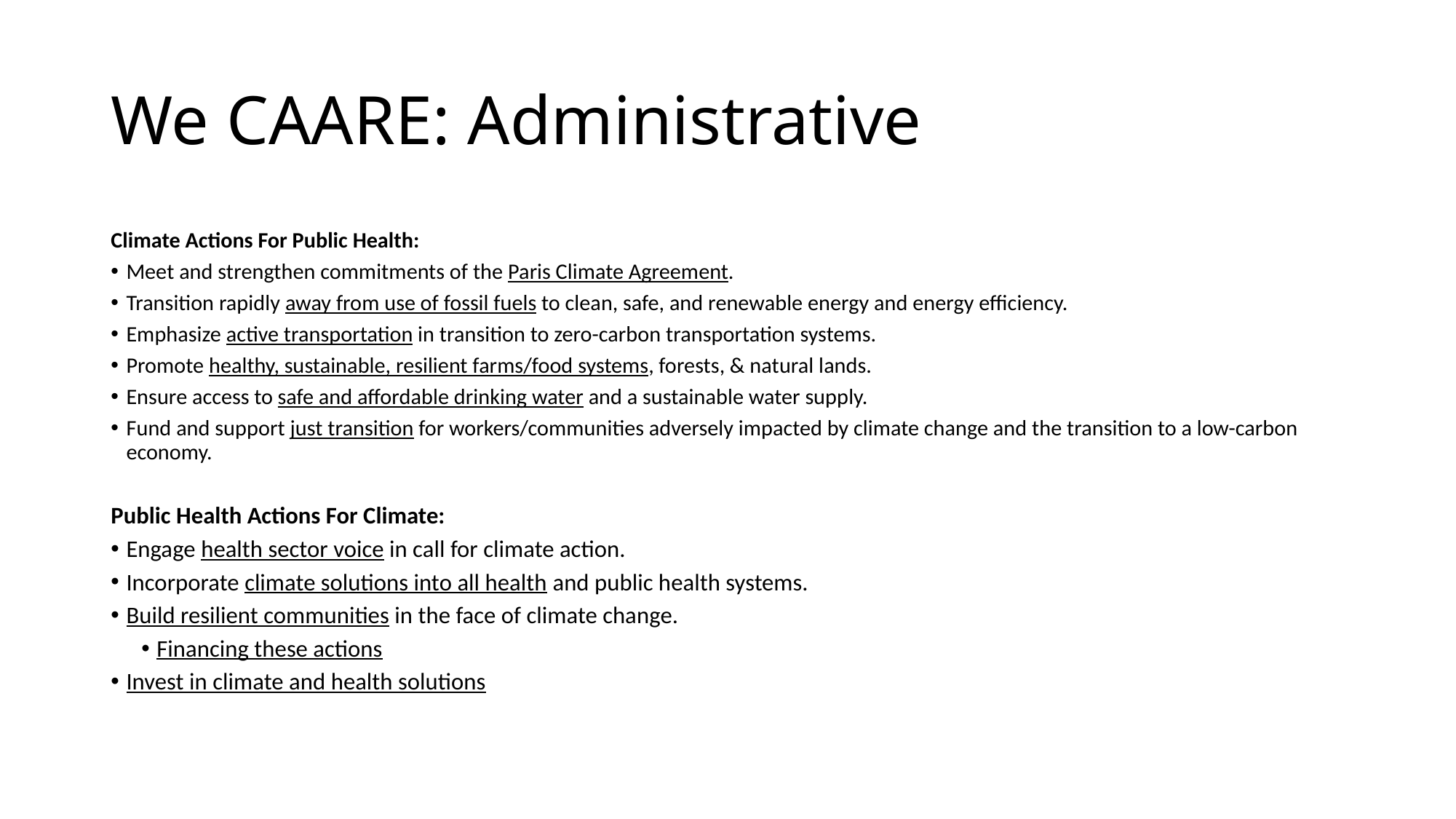

# We CAARE: Administrative
Climate Actions For Public Health:
Meet and strengthen commitments of the Paris Climate Agreement.
Transition rapidly away from use of fossil fuels to clean, safe, and renewable energy and energy efficiency.
Emphasize active transportation in transition to zero-carbon transportation systems.
Promote healthy, sustainable, resilient farms/food systems, forests, & natural lands.
Ensure access to safe and affordable drinking water and a sustainable water supply.
Fund and support just transition for workers/communities adversely impacted by climate change and the transition to a low-carbon economy.
Public Health Actions For Climate:
Engage health sector voice in call for climate action.
Incorporate climate solutions into all health and public health systems.
Build resilient communities in the face of climate change.
Financing these actions
Invest in climate and health solutions

## Slide 24
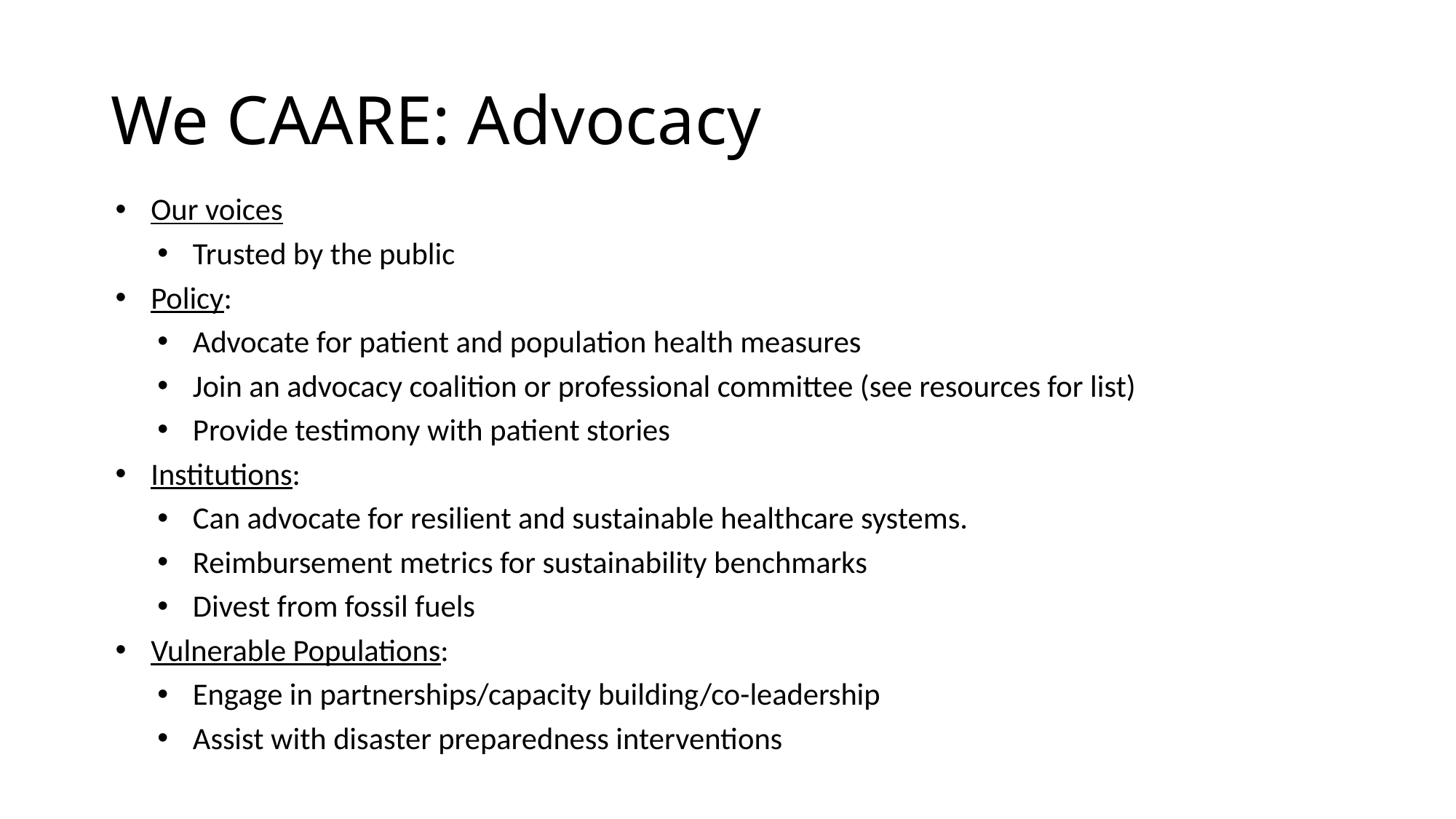

# We CAARE: Advocacy
Our voices
Trusted by the public
Policy:
Advocate for patient and population health measures
Join an advocacy coalition or professional committee (see resources for list)
Provide testimony with patient stories
Institutions:
Can advocate for resilient and sustainable healthcare systems.
Reimbursement metrics for sustainability benchmarks
Divest from fossil fuels
Vulnerable Populations:
Engage in partnerships/capacity building/co-leadership
Assist with disaster preparedness interventions

## Slide 25
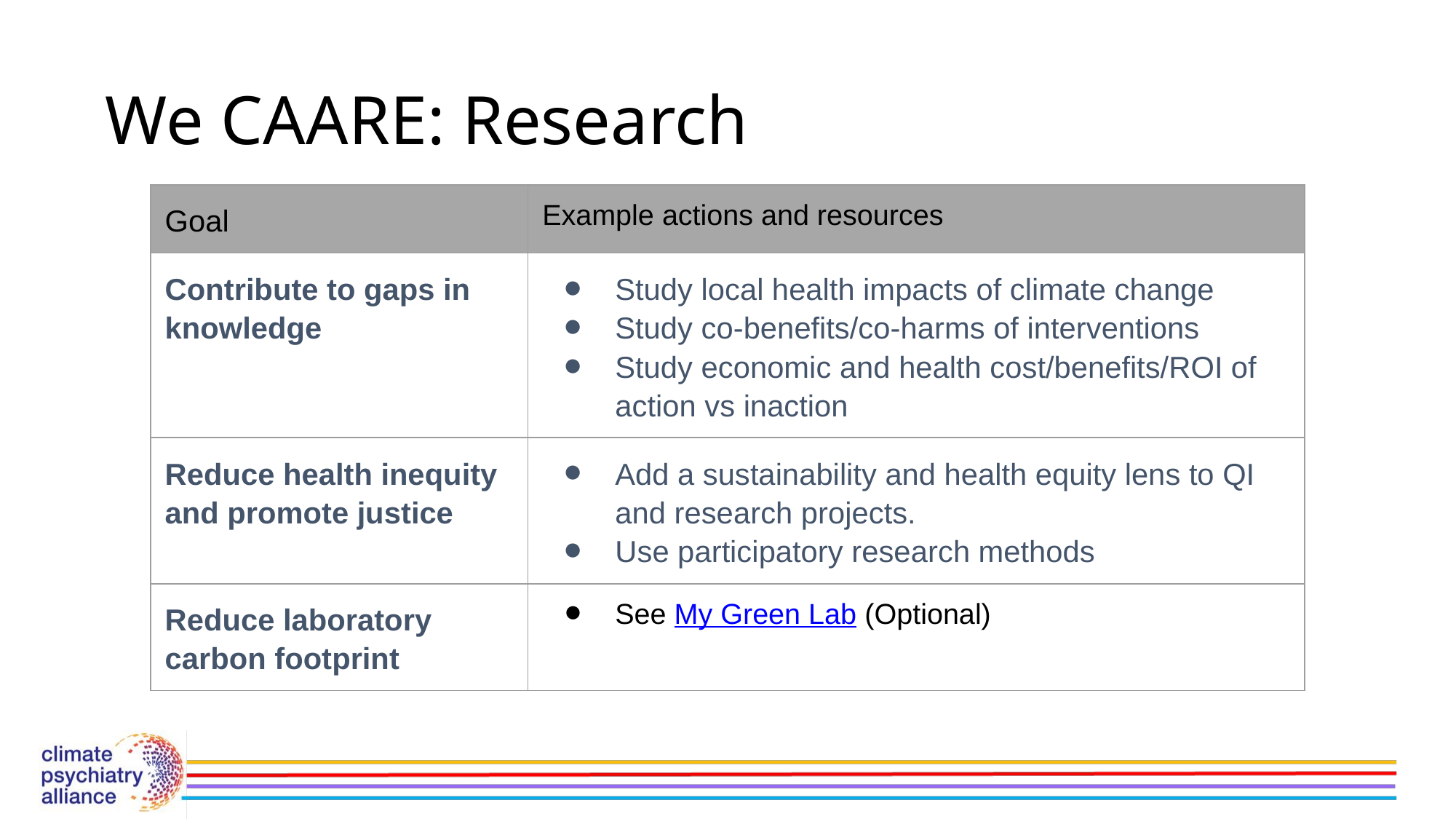

# We CAARE: Research
| Goal | Example actions and resources |
| --- | --- |
| Contribute to gaps in knowledge | Study local health impacts of climate change Study co-benefits/co-harms of interventions Study economic and health cost/benefits/ROI of action vs inaction |
| Reduce health inequity and promote justice | Add a sustainability and health equity lens to QI and research projects. Use participatory research methods |
| Reduce laboratory carbon footprint | See My Green Lab (Optional) |

## Slide 26
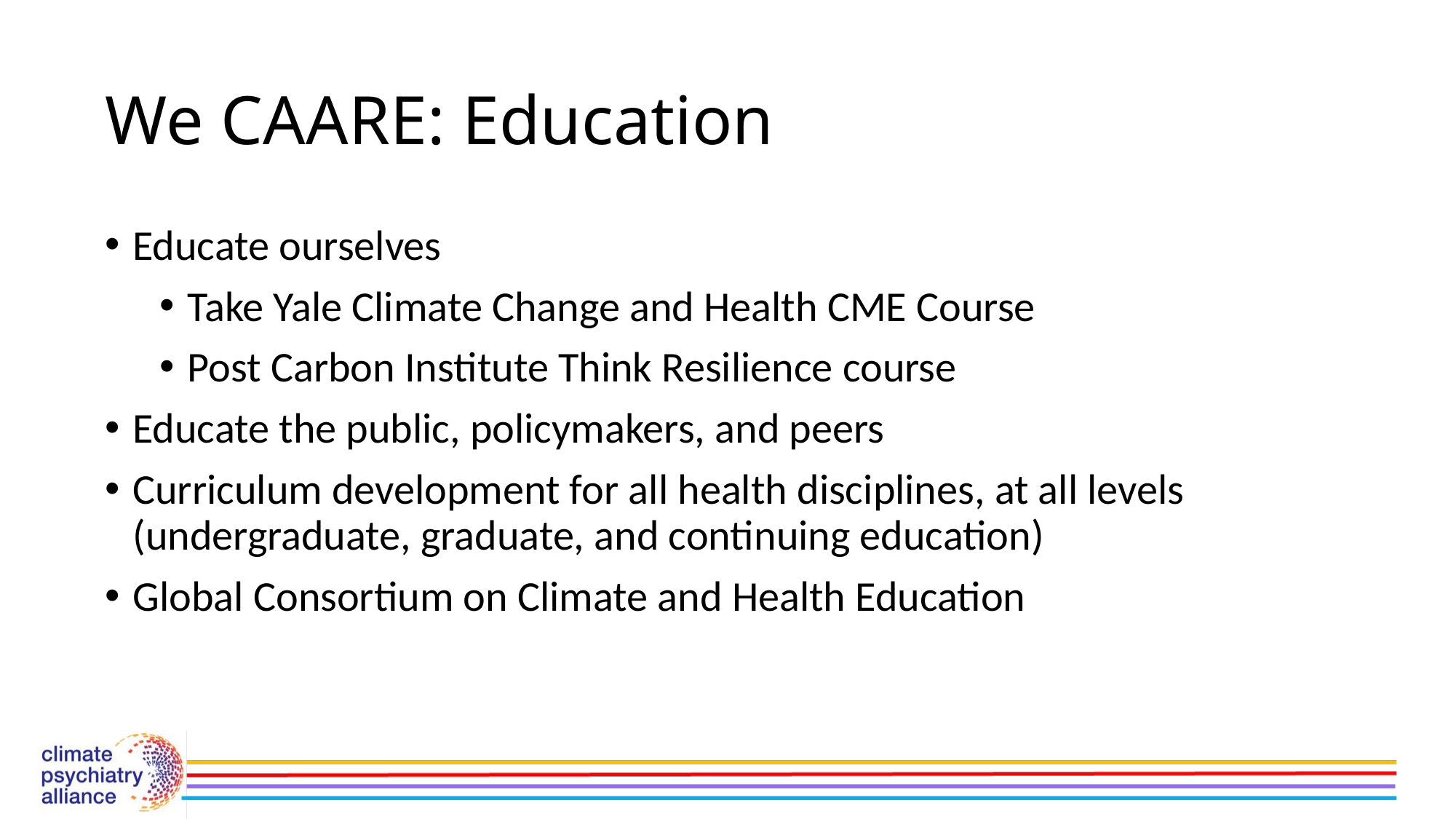

# We CAARE: Education
Educate ourselves
Take Yale Climate Change and Health CME Course
Post Carbon Institute Think Resilience course
Educate the public, policymakers, and peers
Curriculum development for all health disciplines, at all levels (undergraduate, graduate, and continuing education)
Global Consortium on Climate and Health Education

## Slide 27
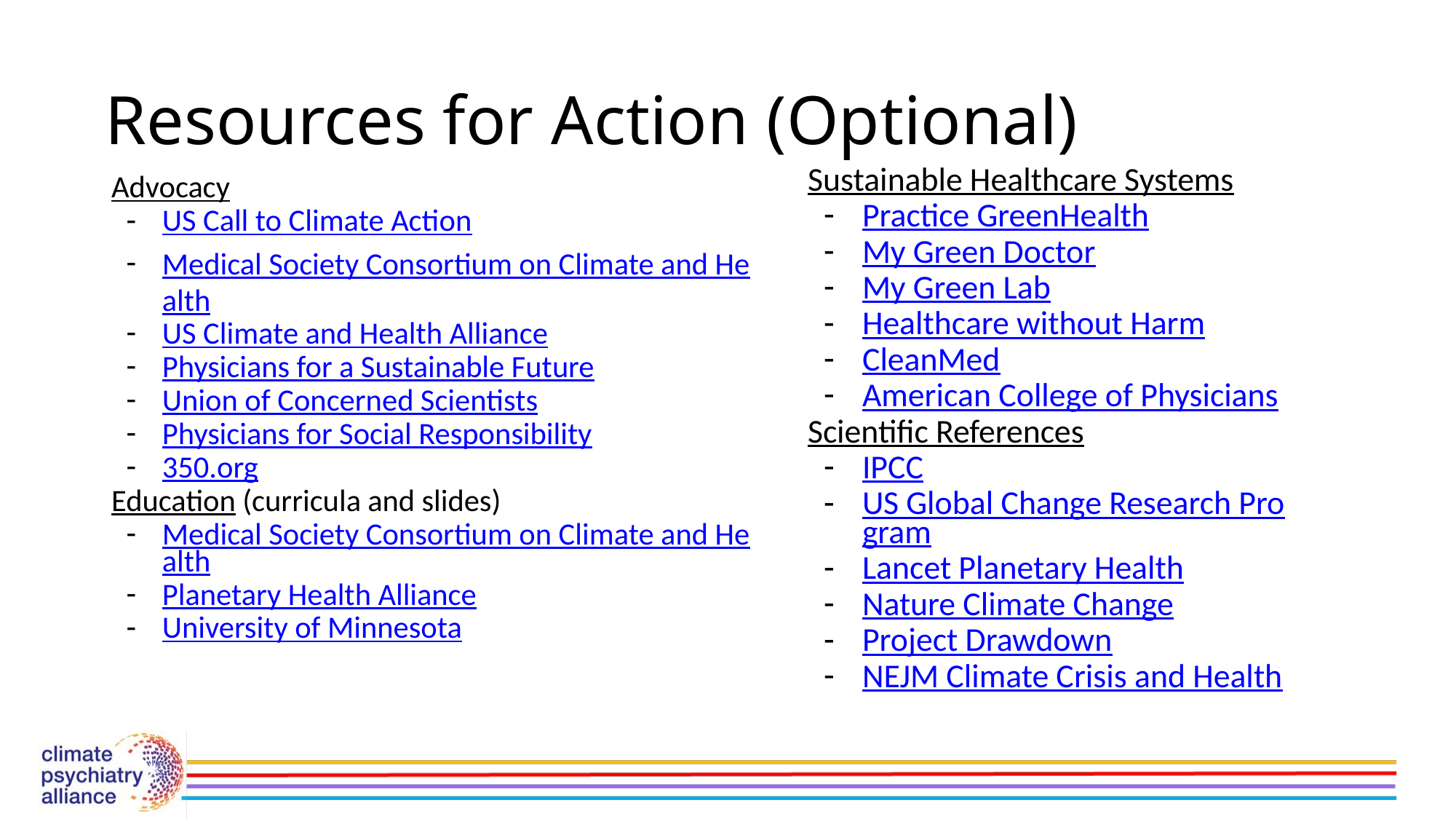

# Resources for Action (Optional)
Sustainable Healthcare Systems
Practice GreenHealth
My Green Doctor
My Green Lab
Healthcare without Harm
CleanMed
American College of Physicians
Scientific References
IPCC
US Global Change Research Program
Lancet Planetary Health
Nature Climate Change
Project Drawdown
NEJM Climate Crisis and Health
Advocacy
US Call to Climate Action
Medical Society Consortium on Climate and Health
US Climate and Health Alliance
Physicians for a Sustainable Future
Union of Concerned Scientists
Physicians for Social Responsibility
350.org
Education (curricula and slides)
Medical Society Consortium on Climate and Health
Planetary Health Alliance
University of Minnesota

## Slide 28
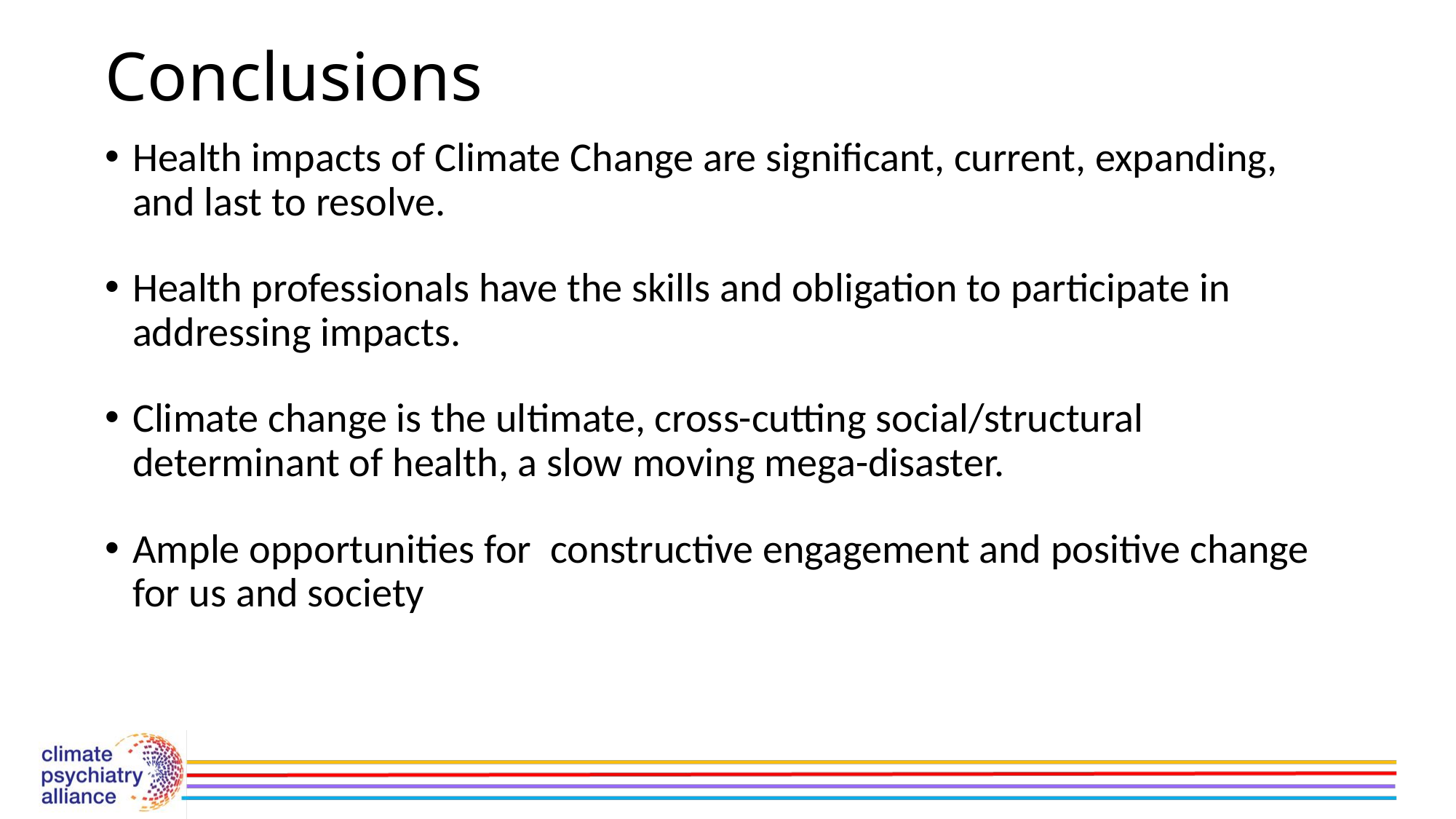

# Conclusions
Health impacts of Climate Change are significant, current, expanding, and last to resolve.
Health professionals have the skills and obligation to participate in addressing impacts.
Climate change is the ultimate, cross-cutting social/structural determinant of health, a slow moving mega-disaster.
Ample opportunities for constructive engagement and positive change for us and society

## Slide 29
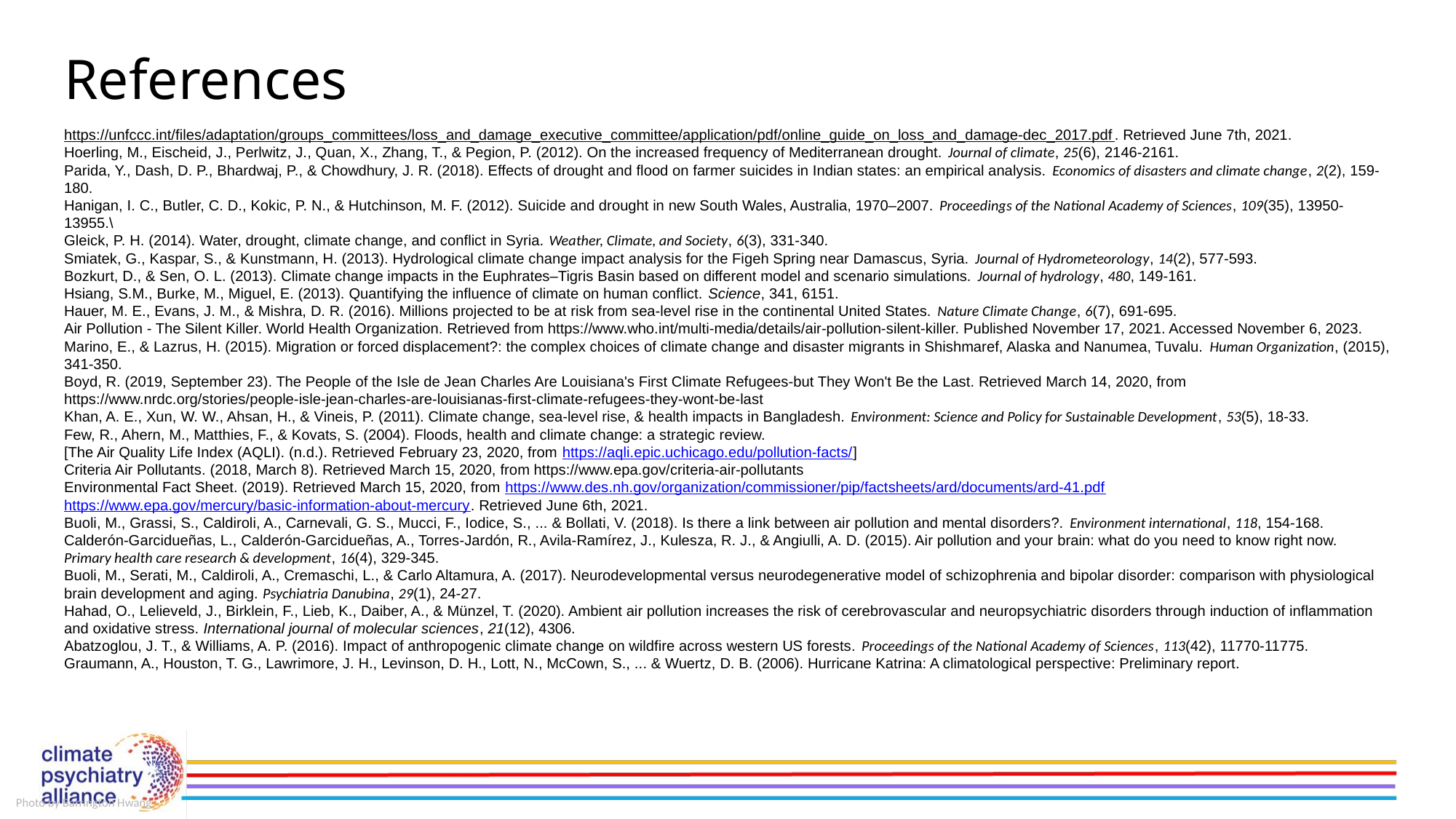

# References
https://unfccc.int/files/adaptation/groups_committees/loss_and_damage_executive_committee/application/pdf/online_guide_on_loss_and_damage-dec_2017.pdf. Retrieved June 7th, 2021.
Hoerling, M., Eischeid, J., Perlwitz, J., Quan, X., Zhang, T., & Pegion, P. (2012). On the increased frequency of Mediterranean drought. Journal of climate, 25(6), 2146-2161.
Parida, Y., Dash, D. P., Bhardwaj, P., & Chowdhury, J. R. (2018). Effects of drought and flood on farmer suicides in Indian states: an empirical analysis. Economics of disasters and climate change, 2(2), 159-180.
Hanigan, I. C., Butler, C. D., Kokic, P. N., & Hutchinson, M. F. (2012). Suicide and drought in new South Wales, Australia, 1970–2007. Proceedings of the National Academy of Sciences, 109(35), 13950-13955.\
Gleick, P. H. (2014). Water, drought, climate change, and conflict in Syria. Weather, Climate, and Society, 6(3), 331-340.
Smiatek, G., Kaspar, S., & Kunstmann, H. (2013). Hydrological climate change impact analysis for the Figeh Spring near Damascus, Syria. Journal of Hydrometeorology, 14(2), 577-593.
Bozkurt, D., & Sen, O. L. (2013). Climate change impacts in the Euphrates–Tigris Basin based on different model and scenario simulations. Journal of hydrology, 480, 149-161.
Hsiang, S.M., Burke, M., Miguel, E. (2013). Quantifying the influence of climate on human conflict. Science, 341, 6151.
Hauer, M. E., Evans, J. M., & Mishra, D. R. (2016). Millions projected to be at risk from sea-level rise in the continental United States. Nature Climate Change, 6(7), 691-695.
Air Pollution - The Silent Killer. World Health Organization. Retrieved from https://www.who.int/multi-media/details/air-pollution-silent-killer. Published November 17, 2021. Accessed November 6, 2023.
Marino, E., & Lazrus, H. (2015). Migration or forced displacement?: the complex choices of climate change and disaster migrants in Shishmaref, Alaska and Nanumea, Tuvalu. Human Organization, (2015), 341-350.
Boyd, R. (2019, September 23). The People of the Isle de Jean Charles Are Louisiana's First Climate Refugees-but They Won't Be the Last. Retrieved March 14, 2020, from https://www.nrdc.org/stories/people-isle-jean-charles-are-louisianas-first-climate-refugees-they-wont-be-last
Khan, A. E., Xun, W. W., Ahsan, H., & Vineis, P. (2011). Climate change, sea-level rise, & health impacts in Bangladesh. Environment: Science and Policy for Sustainable Development, 53(5), 18-33.
Few, R., Ahern, M., Matthies, F., & Kovats, S. (2004). Floods, health and climate change: a strategic review.
[The Air Quality Life Index (AQLI). (n.d.). Retrieved February 23, 2020, from https://aqli.epic.uchicago.edu/pollution-facts/]
Criteria Air Pollutants. (2018, March 8). Retrieved March 15, 2020, from https://www.epa.gov/criteria-air-pollutants
Environmental Fact Sheet. (2019). Retrieved March 15, 2020, from https://www.des.nh.gov/organization/commissioner/pip/factsheets/ard/documents/ard-41.pdf
https://www.epa.gov/mercury/basic-information-about-mercury. Retrieved June 6th, 2021.
Buoli, M., Grassi, S., Caldiroli, A., Carnevali, G. S., Mucci, F., Iodice, S., ... & Bollati, V. (2018). Is there a link between air pollution and mental disorders?. Environment international, 118, 154-168.
Calderón-Garcidueñas, L., Calderón-Garcidueñas, A., Torres-Jardón, R., Avila-Ramírez, J., Kulesza, R. J., & Angiulli, A. D. (2015). Air pollution and your brain: what do you need to know right now. Primary health care research & development, 16(4), 329-345.
Buoli, M., Serati, M., Caldiroli, A., Cremaschi, L., & Carlo Altamura, A. (2017). Neurodevelopmental versus neurodegenerative model of schizophrenia and bipolar disorder: comparison with physiological brain development and aging. Psychiatria Danubina, 29(1), 24-27.
Hahad, O., Lelieveld, J., Birklein, F., Lieb, K., Daiber, A., & Münzel, T. (2020). Ambient air pollution increases the risk of cerebrovascular and neuropsychiatric disorders through induction of inflammation and oxidative stress. International journal of molecular sciences, 21(12), 4306.
Abatzoglou, J. T., & Williams, A. P. (2016). Impact of anthropogenic climate change on wildfire across western US forests. Proceedings of the National Academy of Sciences, 113(42), 11770-11775.
Graumann, A., Houston, T. G., Lawrimore, J. H., Levinson, D. H., Lott, N., McCown, S., ... & Wuertz, D. B. (2006). Hurricane Katrina: A climatological perspective: Preliminary report.
Photo by Barrington Hwang

## Slide 30
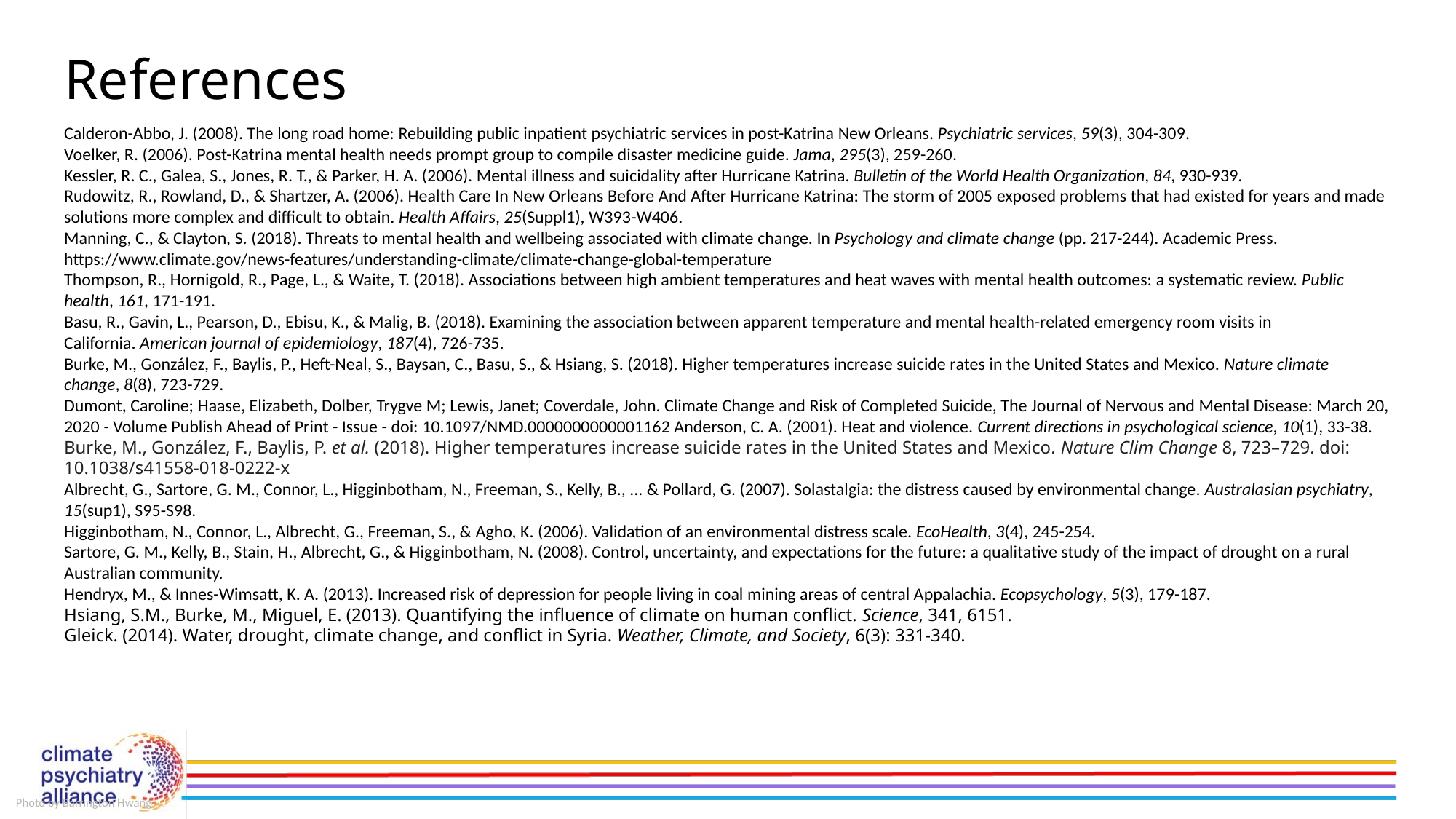

# References
Calderon-Abbo, J. (2008). The long road home: Rebuilding public inpatient psychiatric services in post-Katrina New Orleans. Psychiatric services, 59(3), 304-309.
Voelker, R. (2006). Post-Katrina mental health needs prompt group to compile disaster medicine guide. Jama, 295(3), 259-260.
Kessler, R. C., Galea, S., Jones, R. T., & Parker, H. A. (2006). Mental illness and suicidality after Hurricane Katrina. Bulletin of the World Health Organization, 84, 930-939.
Rudowitz, R., Rowland, D., & Shartzer, A. (2006). Health Care In New Orleans Before And After Hurricane Katrina: The storm of 2005 exposed problems that had existed for years and made solutions more complex and difficult to obtain. Health Affairs, 25(Suppl1), W393-W406.
Manning, C., & Clayton, S. (2018). Threats to mental health and wellbeing associated with climate change. In Psychology and climate change (pp. 217-244). Academic Press.
https://www.climate.gov/news-features/understanding-climate/climate-change-global-temperature
Thompson, R., Hornigold, R., Page, L., & Waite, T. (2018). Associations between high ambient temperatures and heat waves with mental health outcomes: a systematic review. Public health, 161, 171-191.
Basu, R., Gavin, L., Pearson, D., Ebisu, K., & Malig, B. (2018). Examining the association between apparent temperature and mental health-related emergency room visits in California. American journal of epidemiology, 187(4), 726-735.
Burke, M., González, F., Baylis, P., Heft-Neal, S., Baysan, C., Basu, S., & Hsiang, S. (2018). Higher temperatures increase suicide rates in the United States and Mexico. Nature climate change, 8(8), 723-729.
Dumont, Caroline; Haase, Elizabeth, Dolber, Trygve M; Lewis, Janet; Coverdale, John. Climate Change and Risk of Completed Suicide, The Journal of Nervous and Mental Disease: March 20, 2020 - Volume Publish Ahead of Print - Issue - doi: 10.1097/NMD.0000000000001162 Anderson, C. A. (2001). Heat and violence. Current directions in psychological science, 10(1), 33-38.
Burke, M., González, F., Baylis, P. et al. (2018). Higher temperatures increase suicide rates in the United States and Mexico. Nature Clim Change 8, 723–729. doi: 10.1038/s41558-018-0222-x
Albrecht, G., Sartore, G. M., Connor, L., Higginbotham, N., Freeman, S., Kelly, B., ... & Pollard, G. (2007). Solastalgia: the distress caused by environmental change. Australasian psychiatry, 15(sup1), S95-S98.
Higginbotham, N., Connor, L., Albrecht, G., Freeman, S., & Agho, K. (2006). Validation of an environmental distress scale. EcoHealth, 3(4), 245-254.
Sartore, G. M., Kelly, B., Stain, H., Albrecht, G., & Higginbotham, N. (2008). Control, uncertainty, and expectations for the future: a qualitative study of the impact of drought on a rural Australian community.
Hendryx, M., & Innes-Wimsatt, K. A. (2013). Increased risk of depression for people living in coal mining areas of central Appalachia. Ecopsychology, 5(3), 179-187.
Hsiang, S.M., Burke, M., Miguel, E. (2013). Quantifying the influence of climate on human conflict. Science, 341, 6151.
Gleick. (2014). Water, drought, climate change, and conflict in Syria. Weather, Climate, and Society, 6(3): 331-340.
Photo by Barrington Hwang

## Slide 31
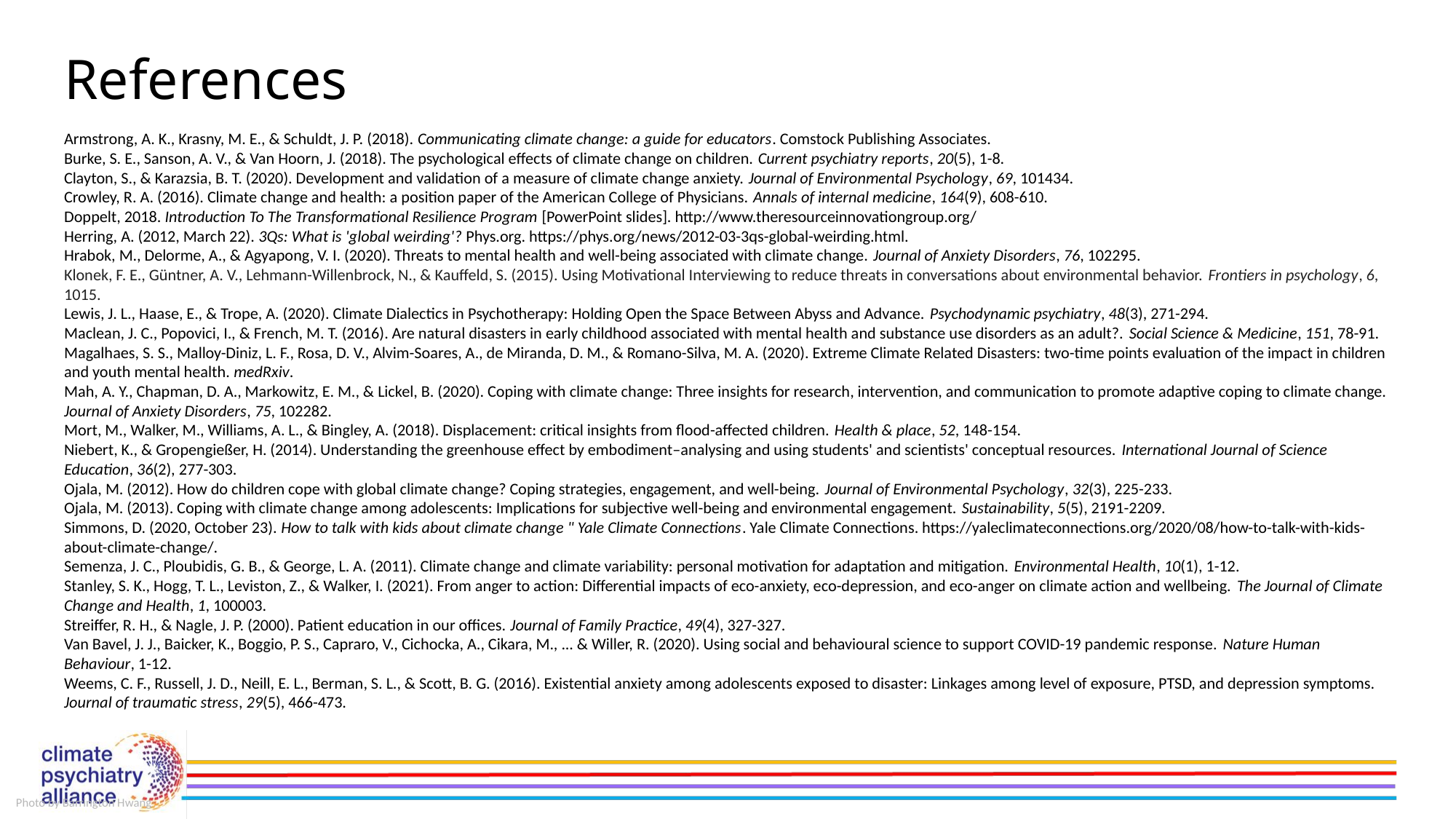

# References
Armstrong, A. K., Krasny, M. E., & Schuldt, J. P. (2018). Communicating climate change: a guide for educators. Comstock Publishing Associates.
Burke, S. E., Sanson, A. V., & Van Hoorn, J. (2018). The psychological effects of climate change on children. Current psychiatry reports, 20(5), 1-8.
Clayton, S., & Karazsia, B. T. (2020). Development and validation of a measure of climate change anxiety. Journal of Environmental Psychology, 69, 101434.
Crowley, R. A. (2016). Climate change and health: a position paper of the American College of Physicians. Annals of internal medicine, 164(9), 608-610.
Doppelt, 2018. Introduction To The Transformational Resilience Program [PowerPoint slides]. http://www.theresourceinnovationgroup.org/
Herring, A. (2012, March 22). 3Qs: What is 'global weirding'? Phys.org. https://phys.org/news/2012-03-3qs-global-weirding.html.
Hrabok, M., Delorme, A., & Agyapong, V. I. (2020). Threats to mental health and well-being associated with climate change. Journal of Anxiety Disorders, 76, 102295.
Klonek, F. E., Güntner, A. V., Lehmann-Willenbrock, N., & Kauffeld, S. (2015). Using Motivational Interviewing to reduce threats in conversations about environmental behavior. Frontiers in psychology, 6, 1015.
Lewis, J. L., Haase, E., & Trope, A. (2020). Climate Dialectics in Psychotherapy: Holding Open the Space Between Abyss and Advance. Psychodynamic psychiatry, 48(3), 271-294.
Maclean, J. C., Popovici, I., & French, M. T. (2016). Are natural disasters in early childhood associated with mental health and substance use disorders as an adult?. Social Science & Medicine, 151, 78-91.
Magalhaes, S. S., Malloy-Diniz, L. F., Rosa, D. V., Alvim-Soares, A., de Miranda, D. M., & Romano-Silva, M. A. (2020). Extreme Climate Related Disasters: two-time points evaluation of the impact in children and youth mental health. medRxiv.
Mah, A. Y., Chapman, D. A., Markowitz, E. M., & Lickel, B. (2020). Coping with climate change: Three insights for research, intervention, and communication to promote adaptive coping to climate change. Journal of Anxiety Disorders, 75, 102282.
Mort, M., Walker, M., Williams, A. L., & Bingley, A. (2018). Displacement: critical insights from flood-affected children. Health & place, 52, 148-154.
Niebert, K., & Gropengießer, H. (2014). Understanding the greenhouse effect by embodiment–analysing and using students' and scientists' conceptual resources. International Journal of Science Education, 36(2), 277-303.
Ojala, M. (2012). How do children cope with global climate change? Coping strategies, engagement, and well-being. Journal of Environmental Psychology, 32(3), 225-233.
Ojala, M. (2013). Coping with climate change among adolescents: Implications for subjective well-being and environmental engagement. Sustainability, 5(5), 2191-2209.
Simmons, D. (2020, October 23). How to talk with kids about climate change " Yale Climate Connections. Yale Climate Connections. https://yaleclimateconnections.org/2020/08/how-to-talk-with-kids-about-climate-change/.
Semenza, J. C., Ploubidis, G. B., & George, L. A. (2011). Climate change and climate variability: personal motivation for adaptation and mitigation. Environmental Health, 10(1), 1-12.
Stanley, S. K., Hogg, T. L., Leviston, Z., & Walker, I. (2021). From anger to action: Differential impacts of eco-anxiety, eco-depression, and eco-anger on climate action and wellbeing. The Journal of Climate Change and Health, 1, 100003.
Streiffer, R. H., & Nagle, J. P. (2000). Patient education in our offices. Journal of Family Practice, 49(4), 327-327.
Van Bavel, J. J., Baicker, K., Boggio, P. S., Capraro, V., Cichocka, A., Cikara, M., ... & Willer, R. (2020). Using social and behavioural science to support COVID-19 pandemic response. Nature Human Behaviour, 1-12.
Weems, C. F., Russell, J. D., Neill, E. L., Berman, S. L., & Scott, B. G. (2016). Existential anxiety among adolescents exposed to disaster: Linkages among level of exposure, PTSD, and depression symptoms. Journal of traumatic stress, 29(5), 466-473.
Photo by Barrington Hwang
