# Supplementary material for: Climate Change and Mental Health: An Interactive Educational Session
Source: MedEdPORTAL. 2024 Apr 19;20:11418. doi: 10.15766/mep_2374-8265.11418 (PMC11026302; doi:10.15766/mep_2374-8265.11418)
Supplement: Supplementary file 1 — Session Presentation.pptxFacilitator Guide.docxPostsession Resources for Students.docxPre- and Postsession Survey.docx [file mep_2374-8265.11418-s001.zip › C. Postsession Resources for Students.docx]

**Post Session Resources for Students**

We CAARE: Clinical

- Plant Based Diet
- Active Commute
- Green Meds and Anesthetics
- Green Procedures
- Reduce Waste
- Protect Vulnerable Patients
- Patient Communication
- Resources (Optional): [Practice GreenHealth](https://practicegreenhealth.org/), [My Green Doctor](https://www.mygreendoctor.org/), [Healthcare without Harm](https://noharm.org/), [CleanMed](https://cleanmed.org/)

Patient Materials (Optional)

- [National Environmental Health Association](https://www.neha.org/climate-change)
- [NEJM Interactive Climate Crisis Module](https://www.nejm.org/doi/full/10.1056/NEJMp1906035)
- [Climate Psychiatry Alliance](https://www.climatepsychiatry.org/resources-to-mitigate-climate-distress)

We CAARE: Administrative

**Climate Actions For Public Health:**

- Meet and strengthen commitments of the Paris Climate Agreement.
- Transition rapidly away from use of fossil fuels to clean, safe, and renewable energy and energy efficiency.
- Emphasize active transportation in transition to zero-carbon transportation systems.
- Promote healthy, sustainable, resilient farms/food systems, forests, & natural lands.
- Ensure access to safe and affordable drinking water and a sustainable water supply.
- Fund and support just transition for workers/communities adversely impacted by climate change and the transition to a low-carbon economy.

**Public Health Actions For Climate:**

- Engage health sector voice in call for climate action.
- Incorporate climate solutions into all health and public health systems.
- Build resilient communities in the face of climate change.
  - Financing these actions
- Invest in climate and health solutions.

We CAARE: Advocacy

- Our voices
  - Trusted by the public
- Policy:
  - Advocate for patient and population health measures
  - Join an advocacy coalition or professional committee (see resources for list)
  - Provide testimony with patient stories
- Institutions:
  - Can advocate for resilient and sustainable healthcare systems.
  - Reimbursement metrics for sustainability benchmarks
  - Divest from fossil fuels
- Vulnerable Populations:
  - Engage in partnerships/capacity building/co-leadership.
  - Assist with disaster preparedness interventions.

We CAARE: Research

| Goal | Example actions and resources |
| --- | --- |
| **Contribute to gaps in knowledge** | - Study local health impacts of climate change - Study co-benefits/co-harms of interventions - Study economic and health cost/benefits/ROI of action vs inaction |
| **Reduce health inequity and promote justice** | - Add a sustainability and health equity lens to QI and research projects. - Use participatory research methods |
| **Reduce laboratory carbon footprint** | - See [My Green Lab](https://www.mygreenlab.org/) (Optional) |

We CAARE: Education (Optional)

- Educate ourselves
  - Take [Yale Climate Change and Health CME Course](https://publichealth.yale.edu/cchcert/program/cme/)
  - Post Carbon Institute [Think Resilience](https://education.resilience.org/product/self-directed-course/) course.
- Educate the public, policymakers, and peers.
- Curriculum development for all health disciplines, at all levels (undergraduate, graduate, and continuing education)
- [Global Consortium on Climate and Health Education](https://www.publichealth.columbia.edu/research/programs/global-consortium-climate-health-education)
- [Medical Society Consortium on Climate and Health](https://medsocietiesforclimatehealth.org/)

Resources for Action (Optional)

Advocacy

- [US Call to Climate Action](https://climatehealthaction.org/media/cta_docs/US_Call_to_Action.pdf)
- [Medical Society Consortium on Climate and Health](https://medsocietiesforclimatehealth.org/)
- [US Climate and Health Alliance](http://usclimateandhealthalliance.org/)
- [Physicians for a Sustainable Future](https://www.facebook.com/groups/PhysiciansForASustainableFuture/)
- [Union of Concerned Scientists](https://www.ucsusa.org/)
- [Physicians for Social Responsibility](https://www.psr.org/)
- [350.org](https://350.org/)

Education (curricula and slides)

- [Medical Society Consortium on Climate and Health](https://medsocietiesforclimatehealth.org/educate/)
- [Planetary Health Alliance](https://www.planetaryhealthalliance.org/education)
- [University of Minnesota](https://globalhealthcenter.umn.edu/education/climatehealth)

Sustainable Healthcare Systems

- [Practice GreenHealth](https://practicegreenhealth.org/)
- [My Green Doctor](https://www.mygreendoctor.org/)
- [My Green Lab](https://www.mygreenlab.org/)
- [Healthcare without Harm](https://noharm.org/)
- [CleanMed](https://cleanmed.org/)
- [American College of Physicians](https://www.acponline.org/advocacy/advocacy-in-action/climate-change-toolkit)

Scientific References

- [IPCC](https://www.ipcc.ch/)
- [US Global Change Research Program](https://www.globalchange.gov/)
- [Lancet Planetary Health](https://www.thelancet.com/journals/lanplh/home)
- [Nature Climate Change](https://www.nature.com/nclimate/)
- [Project Drawdown](https://www.drawdown.org/)
- [NEJM Climate Crisis and Health](https://www.nejm.org/climate-crisis)
