# Supplementary material for: Climate Change and Mental Health: An Interactive Educational Session
Source: MedEdPORTAL. 2024 Apr 19;20:11418. doi: 10.15766/mep_2374-8265.11418 (PMC11026302; doi:10.15766/mep_2374-8265.11418)
Supplement: Supplementary file 1 — Session Presentation.pptxFacilitator Guide.docxPostsession Resources for Students.docxPre- and Postsession Survey.docx [file mep_2374-8265.11418-s001.zip › D. Pre- and Postsession Survey.docx]

**Pre-Lecture Survey:**

1. I am knowledgeable about climate change’s effects on human physical and mental health.
   1. Strongly agree
   2. Agree
   3. Neutral
   4. Disagree
   5. Strongly disagree
2. I feel comfortable communicating to patients and peers about climate change and human health.
   1. Strongly agree
   2. Agree
   3. Neutral
   4. Disagree
   5. Strongly disagree
3. I am prepared to take actions in my individual and collective practice as a health professional to limit the effects of climate change on human health.
   1. Strongly agree
   2. Agree
   3. Neutral
   4. Disagree
   5. Strongly disagree
4. I am aware of the available resources on climate change and human health to support my actions as a health professional.
   1. Strongly agree
   2. Agree
   3. Neutral
   4. Disagree
   5. Strongly disagree

**Post-Lecture Survey**

1. I am knowledgeable about climate change’s effects on human physical and mental health.
   1. Strongly agree
   2. Agree
   3. Neutral
   4. Disagree
   5. Strongly disagree
2. I feel comfortable communicating to patients and peers about climate change and human health.
   1. Strongly agree
   2. Agree
   3. Neutral
   4. Disagree
   5. Strongly disagree
3. I am prepared to take actions in my individual and collective practice as a health professional to limit the effects of climate change on human health.
   1. Strongly agree
   2. Agree
   3. Neutral
   4. Disagree
   5. Strongly disagree
4. I am aware of the available resources on climate change and human health to support my actions as a health professional.
   1. Strongly agree
   2. Agree
   3. Neutral
   4. Disagree
   5. Strongly disagree
5. What is the single most important thing you learned today? (short answer text)
6. Comments for improvement: (short answer text)
