# Supplementary material for: Climate Change and Mental Health: An Interactive Educational Session
Source: MedEdPORTAL. 2024 Apr 19;20:11418. doi: 10.15766/mep_2374-8265.11418 (PMC11026302; doi:10.15766/mep_2374-8265.11418)
Supplement: Supplementary file 1 — Session Presentation.pptxFacilitator Guide.docxPostsession Resources for Students.docxPre- and Postsession Survey.docx [file mep_2374-8265.11418-s001.zip › B. Facilitator Guide.docx]

**Appendix B: Facilitator Guide**

**Climate Change and Mental Health: An Interactive Educational Session**

**Resource Files Included in Submission:**

Appendix A: Session Presentation.pptx

Appendix B: Facilitator Guide.docx

Appendix C: Postsession Resources for Students.docx

Appendix D: Pre- and Postsession Survey.docx

**Description:**

Following an introduction to the topic and learning objectives, this interactive educational session begins with a facilitator-led discussion on climate change and its impact on health to assess baseline knowledge and spur initial engagement on the topic. This discussion is followed by a mixture of didactic-based slides and questions highlighting various impacts of climate change on mental health. Open-ended discussion and knowledge-based questions can be utilized in either an open forum or anonymous survey software formats. At the end of the session, a resource sheet for more information on interventions, education, and scientific references should be provided to students.

This session can be delivered without any advanced preparation or may be paired with a brief overview of climate change’s impacts on human health, as listed in the suggested resource below.

**Intended Audience:**

This resource was designed for medical students on their psychiatry clinical clerkship but may be adapted for other students in medical education, particularly those in mental health blocks.

**Educational Objectives:**

By the end of this session, learners will be able to:

- Recognize psychiatric conditions and other mental health (MH) impacts that emerge from and are affected by the climate crisis (CC).
- Consider methods of climate communication in the clinical setting.
- Understand roles that health professionals should perform in facilitating resilience and pro-environmental behaviors.
- Become aware of available resources to facilitate such public health and mental health activities.

**Conceptual Background:**

This interactive educational session was authored by John Sullenbarger, M.D., assistant residency training program director at Wright State University, steering committee member of the Climate Psychiatry Alliance (CPA), in collaboration with CPA and Bethany Harper, M.D., director of medical student education in psychiatry at Wright State University. It was designed to be implemented in the third-year medical school psychiatry clerkship, correlating to systems-based practice curriculum core competencies. Interactive open-ended discussion and knowledge-based questions were created and interspersed with didactic slides to encourage active participation while also providing ample time for adequate breadth and depth information of a novel topic in the medical school’s curriculum. While the majority of the time is spent on the mental health impacts of climate change, the suggested preparatory materials, corresponding interventions, and resource sheet is applicable to all fields of medicine given rotating medical students have not formally decided on a specialty during this clerkship.

Climate change is a progressively worsening global crisis that will impact healthcare delivery, individual patients, and society during this century. Consequently, students and physicians will encounter patients and healthcare issues during their clinical rotations and medical career stemming from climate change. C*an say: This interactive educational session was devised to address the gap in most medical school curricula on this topic.* There is a dearth in most medical school curricula on this topic, thus this interactive educational session was devised to start filling this gap.

**Implementation:**

Equipment needed: This interactive educational session can be administered with only a computer and projector for the PowerPoint presentation. If desired, an online anonymous polling (e.g. polleverywhere.com or surveymonkey.com) service can be utilized for the discussion and multiple choice questions which can encourage greater participation.

Some photos were removed in order to protect copyrighted images while others were included with references in the submitted PowerPoint. Additional images can be added or substituted to enhance the visual engagement of the presentation.

Length of session: This educational session was intended to be delivered over 50-60 minutes with the last 10 minutes preferably utilized for discussion, questions, and feedback.

Suggested Preparatory Materials:

1. *The Climate Crisis - Health and Care Delivery*: An online, free interactive module from The New England Journal of Medicine (NEJM). Clicking the ‘play’ button on the image will launch the interactive portion that explains basic climate change science, climate drivers, how humans are exposed to climate change, and the impacts on individual health and healthcare systems. The impacts on individual health are organized by organ systems, while the impacts on healthcare systems are also balanced with corresponding interventions.

**Module Discussion Questions:**

The interactive educational session starts with an open-ended discussion about climate change and its impacts on human health, focusing on mental health. Afterwards, the informational slides are interspersed with multiple choice and open-ended questions to gauge knowledge and generate thoughts on the topic. We suggest permitting 2-4 responses depending on the engagement of the group before summarizing their comments and providing the correct answer choice. The students’ answer choices can then be correlated with content of the slides and presenter notes.

Questions and Desired Responses:

*Q1 (slide 3): What is climate change?*

This is a broad question at the beginning of the session that is intended to gauge baseline knowledge of climate change and how it exerts impacts on human health. If the NEJM interactive module was assigned as prework, students should be expected to have at least a rudimentary understanding. Points of interest that should be highlighted include:

- Climate change, as commonly referred to in current times, is the progressive warming of Earth leading to the destabilization of its planetary systems, worsening extreme weather events and slow moving disasters, and more.
- Climate change, as commonly referred to in current times, is caused by humans burning fossil fuels since the industrial revolution.
- Fossil fuel combustion has released greenhouse gases (e.g. CO2, methane, etc.) into the atmosphere, trapping heat on Earth’s surface.
- Warming of the planet due to increased greenhouse gasses has led to positive feedback loops (e.g. more wildfires releasing more greenhouse gasses into the atmosphere, leading to more warming) which accelerates global warming.

*Q2 (slide 3): How does climate change affect human health?*

This question is intended to invoke basic understanding in students that climate change will affect their practice of medicine regardless of what specialty they choose. Again, the NEJM interactive module will enhance the corresponding discussion with this question. Health impacts from climate change that could be highlighted include:

- Increased rates of heart attacks and strokes due to air pollution increasing vascular inflammation.
- Increased incidence of vector-borne and water-borne illnesses due to changing habitats, higher temperatures, and extreme weather events.
- Higher temperatures and CO2 concentrations diminish nutritional yield of many staple crops and worsen seasonal allergies due to higher pollen production.
- Higher temperatures and heat waves are expected to cause increased incidence of heat-related illness, as well as increased renal dysfunction (e.g. renal failure, electrolyte imbalances, and kidney stones).
- Air pollution is expected to cause new respiratory symptoms and exacerbate existing respiratory disease.

*Q3 (slide 3): How does climate change affect mental health?*

Discussion prompted by this question is to serve as a launching point for the remainder of the educational session and responses can be echoed during the didactic slides as well. The facilitator’s response to these comments should commonly include validation as they often allude to each student’s own emotional experience of climate change. There may be numerous different but valid responses by students, some of the most common may include:

- A sense of hopelessness due to the expansive impacts of climate change.
- Anxiety due to wide-ranging and worsening impacts of climate change, or what will be lost as a result of climate change.
- Increased rates of psychiatric disorders (e.g. PTSD, anxiety, depression, etc.) due to climate change-related disasters and associated trauma.

*Q4 (slide 4): What causes the greatest amount of lost life, in terms of decreased life expectancy, in the world?*

The correct answer is C (Air pollution (e.g. air particulates, ground level ozone, etc.)). This is explained further on slide 8 which explains that air pollution causes 1.8 years of average life expectancy lost per person worldwide. This is primarily a result of vascular inflammation precipitating increased rates of heart attacks and strokes.

*Q5 (slide 13): 1 standard deviation above average temperature is strongly correlated with what percentage change in interpersonal and intergroup violence, respectively?*

The correct answer is A (4%, 14%). This question is meant to highlight that suicide and violence rates have been established to rise with increasing temperatures, a particularly important fact for mental health professionals. It can be helpful to point out that rates of suicide and violence also increase with drought, a slow-moving disaster that is also exacerbated by climate change.

*Q7 (slide 18): How can we, as health professionals, contribute to fixing the climate crisis?*

This question demarks a shift in the interactive educational session from talking about the impacts of climate change to now speaking about actions that students and other health professionals can take to help address climate change. This is an important shift as the early portions of this educational session can be ridden with heavy, negative emotions for students and thus the remainder of the session is meant to restore students with a sense of agency as it relates to this topic. There may be numerous responses to this question and they all can be framed within the acronym used on the next slide, ‘We CAARE’, referring to Clinical, Administrative, Advocacy, Research, and Education areas of medical practice.

**Presenter Notes**:

This topic may be novel for many facilitators providing this interactive educational session to their students and includes a large portion of didactic-based slides to deliver important information. To help facilitators fill in knowledge gaps both prior to and during implementation of this session, we have provided presenter notes dictated by the author of the slides to assist delivering the session.

Slide 1 - Introduction: Introduce self and the topic

Slide 2 - Learning Objectives: Read the learning objectives to students

Slide 3 - Discussion Slide: Please see Q1, Q2, Q3 and desired responses above

Slide 4 - Question Slide: Please see Q4 and desired response above

Slide 5 - Slow-Moving Disasters Overview:

“The next slides are dedicated to broad overviews of acute and slow disasters as well as their effects on mental health.

The 2015 Paris Agreement (unfcc.int) summarized eight slow onset threats of climate change that do not show effects immediately. Among these slow onset events are drought, sea level rise, and ecosystem degradation which have more visible impacts on mental health. Air pollution is an additional slow-moving disaster with significant consequences to mental health. The remainder of them do not deserve to be skimmed over and will undoubtedly trickle down to affect human health, but for brevity we will focus on just several.

As we go through the next few slides, I’d like to emphasize the commonality of these disasters - namely people being forced from their homes as climate refugees. This forced migration disrupts people’s social community, identities, and exacerbates conflict.”

Slide 6 - Slow-Moving Disasters: Drought

“Climate change is already influencing droughts by reducing winter rainfall and increasing evapotranspiration (Hoerling et al 2012). The decreased availability of freshwater has a large range of effects, such as exacerbation of fire seasons, dehydration, diminished irrigation. Farmers are one group that take big losses due to water loss, correlating with higher depression rates. There have been documented suicide epidemics in Indian (Parida et al 2018) and Australian (Hanigan et al 2012) farmers associated with droughts there.

And droughts have a big part in worsening conflicts as we’ll talk about in a few slides (Hsiang et al. 2013). That scenario has already been playing out in parts of the world. Severe, prolonged drought and the subsequent economic downturn can place climate refugees fleeing the drought’s effects in urban areas, putting strains on already low-resource areas. This process has played at least a part in conflicts in places like Syria (Gleick 2014).”

Slide 7 - Slow-Moving Disasters: Sea-Level Rise

“Sea Level Rise is a byproduct of climate change due to melting ice sheets and, most importantly, the expansion of the ocean as it warms. Exactly how much it rises is not exact but a 2019 report by UN’s Intergovernmental Panel on Climate Change, or IPCC, found that global mean sea levels will most likely rise between 1 - 3.5 feet by the end of this century. In the U.S., a sea-level rise of 3ft by year 2100 places a projected 4.2 million people at risk of inundation. (Hauer et al. 2016). In the US, we already have refugees from places like Alaska (Marino & Lazrus 2015) and Louisiana (Boyd 2019) due to sea level rise. Some island countries' entire existences are threatened, such as Pacific Ocean island nations and the Maldives - the lowest lying country in the world.

This extent of sea level rise increases the coastal lands risk of shoreline erosion, contamination of freshwater sources, and flooding. Flooding in particular has been associated with increased rates of anxiety, depression, PTSD, irritability, sleeplessness, and suicide (Few et al. 2004).”

Slide 8 - Slow-Moving Disasters: Air Pollution

“Moving on to air pollution. It is commonly known that CO2 levels from fossil fuels are the primary cause of human-induced climate change, but the refining process and burning of fossil fuels also produces many other air pollutants. There are six criteria air pollutants that impact human health managed by the EPA, as noted by the Clean Air Act (Criteria Air Pollutants 2018). All of which are frequent byproducts of fossil fuels (Environmental Fact Sheet 2019). Out of all these air pollutants, particulate matter receives the most attention. When we talk about particulate matter. we are oftentimes referring to PM2.5, the residual matter from combustion that is 2.5 microns or smaller - that size is small enough to get deep into our lungs and alveoli, crossing into the bloodstream and the blood brain barrier.

The Air Quality Life Index (AQLI) is an authority on air pollution, and one of their core findings is that particulate air pollution cuts global life expectancy short by nearly 2.2 years relative to if particulate concentrations were at the levels deemed safe by the WHO. Moreover, 75 percent of the global population, or 5.5 billion people, live in areas where PM_2.5_ exceeds the WHO guideline, making it the greatest cause of lost life in the world cumulatively (AQLI).”

Slide 9 - Slow-Moving Disasters: Air Pollution (cont.)

“Most of that loss of life expectancy is a result of PM2.5’s effects on cardiovascular health such as heart attacks and strokes. That is fairly well known by this point. However, air particulates are also neurotoxic by causing systemic inflammation and related brain oxidative stress (Calderon-Garciduenas et al. 2015; Buoli et al. 2017). The strongest associations being with autism, dementia, and stroke which has numerous psychiatric comorbidities (Buoli et al. 2018; Hahad et al. 2020).”

Slide 10 - Acute Disasters Overview

“Over the next couple slides we will talk about the acute disasters, primarily referring to wildfires and extreme weather events like hurricanes, flooding, derechos, atmospheric rivers, and more. We will also mention direct health impacts of these disasters such as air pollution from wildfires but focus on the indirect health impacts because they are far greater reaching and longer lasting.”

Slide 11 - Acute Disasters: Wildfires

“First, wildfires. Wildfires are becoming more prevalent throughout the world, with greater geographic ranges into places like Siberia, Alaska, and Scandinavia. And higher frequency, intensity, and burned acreage in places where wildfires already occurred regularly like the Western US, Australia, and Brazilian Pantanal wetlands. There are numerous contributing factors: heat, dryness, drought, unhealthy forests due to vector intrusion like pine bark beetles. Climate change directly exacerbates, or at least acts as a threat multiplier, to most of these wildfire contributors.

Numerous recent studies have looked at the association between climate change and wildfires. For instance, a 2016 study showed human-caused climate change doubled the cumulative forest fire area since 1984 in the Western US (Abatzoglou & Williams 2016). In addition to the anxiety and terror when the fires are ongoing, there is a tremendous release of 2.5-micron particulate matter and volatile organic compounds like formaldehyde, CO, nitrous oxide that are associated w/ increased risk of neurocognitive impairment like Alzheimer’s (Leigh 2020).”

Slide 12 - Acute Disasters: Extreme Weather Events

“Next severe storms. Most studies and models have shown that there will be more flooding and other extreme weather events with climate change. This trajectory is regularly reflected in news headlines with severe floods and other storms, like hurricanes.

Hurricane Katrina in 2005 was heavily studied given its historic nature. The most costly disaster in US history with over 106 billion dollars in costs. One of the most striking findings for mental health professionals was that the number of individuals with a serious mental illness increased from 6.1% before Katrina to 11.3% after the disaster (p<.001), and the number with "any mental illness" rose from 15% to 31% (p<.001) (Kessler et al. 2006; Voelker 2006; Rudowitz et al. 2006). This is in the context of the disaster also depleting health resources and staffing, many of whom were also traumatized and grieving, as well as forced closures of psychiatric facilities after the hurricane, limiting access to care just as there are more people needing care (Graumann et al. 2006).”

Slide 13 - Discussion Slide: Please see Q5 and desired response

Slide 14 - Extreme Heat Effects

“Extreme heat is a very important topic for mental health. The ten hottest years on record have been since 2005. And the rate of temperature increase per decade is more rapid than any period observed through geological studies (NOAA).

A systematic review on 35 studies yielded 6 broad mental health categories effected by extreme heat, but the strongest evidence exists for increased suicide and violence risk in high temperatures. A study in 2018 showed a pretty direct correlation between rates of suicide and temperature increases both in the US and Mexico, as shown by the graph on the left. A 1-degree Celsius increase from average temperature is correlated with a rise in suicide rates of 0.7% in the US and 2.1% in Mexico. This occurs across all socio-economic levels (Burke et al. 2018).

As for violence, there are 2.6% more murders and assaults in the US during summer months vs other seasons. A study performed in Columbus, Ohio, is shown in the graphic on the right. This study correlated daily violent crime counts with daily temperature highs in 2007 and revealed a very tight association between the two. Strong data shows that 1 standard deviation above average temperature can cause a 4% increase in interpersonal violence as well as 14% increase in intergroup violence (Hsiang et al. 2013). This obviously has implications for the risk of future human conflicts and associated trauma.

There is also a significant increase in mental-health related admissions and ED visits at higher temperatures (Thompson et al. 2018).”

Slide 15 - Extreme Heat Effects (cont.)

“Psychiatric medications are a particular concern with extreme heat as many interact negatively with the body’s normal temperature regulation (Martin-Latry et al. 2007). Antipsychotics, stimulants, some antidepressants, and anti-Parkinsonian drugs interfere with heat regulation and sweating, the body’s primary mechanism for cooling. Additionally, lithium levels can rise due to dehydration with extreme heat, risking dangerous or toxic blood levels. And signs of heat exhaustion and lithium toxicity can go unnoticed by our patients with impaired insight.

Heat also has significant impacts on cognition as represented by old sayings like, “it's too hot to think.” The science supports this. Increased temperatures can impair measured attention, reaction times, working memory, and school performance; with older aged populations being particularly susceptible (Mazloumi et al. 2014; Hancock et al. 2003; Schlader et al. 2015; Laurent et al. 2018).

And lastly, there is evidence supporting decreased sleep quality, and decreased exercise frequency with increasing temperatures. This has indirect effects on our patients’ moods, anxiety, or might even precipitate mania with diminished sleep (Obradovich et al. 2017; Obradovich & Fowler 2017).”

Slide 16 - Disproportionate Vulnerability

“It’s important to note that none of the things we’ve talked about occur within a silo. The health impacts from climate change are all influenced by an individual’s unique situation and demographics, otherwise known as determinants of health. This is why we say that the climate crisis is a threat multiplier of social determinants of health.

Racial discrimination, socioeconomic status, age group, pregnancy status, preexisting mental and other conditions, and one’s occupation can all place people at more exposure or vulnerability to climate change impacts, invoke barriers to accessing healthcare, and ultimately worsen health outcomes.”

Slide 17 - Climate Distress

“Climate distress encompasses all the fear and grief that comes from the innumerable loss, threats, uncertainty, and instability which come with the climate crisis. There are many different responses to this: sadness, anger, anxiety, hopelessness, denial or disavowal, and more. Many of these reactions should NOT be considered pathological, but within the range of normal emotional responses to a realistic problem. So we wouldn’t treat these people with anti-anxiety medications, but perhaps they may benefit from individual therapy, referrals to community support, or encouraging involvement in local climate change-related groups or initiatives to build community support and a sense of agency.”

Slide 18 - Discussion Slide: Please see Q7 and desired response

Slide 19 - Agenda for Health Professionals and Systems: We CAARE

“Now that we’ve talked about some of the mental health impacts of climate change, I thought we would make the rest of this talk more generalizable as you may not all become psychiatrists but you will all become physicians. And as physicians, we do have an obligation to address issues that will impact our patients' health, including climate change. So, one acronym being used often is ‘ We CAARE’ to describe areas of practice we can act as individual healthcare professionals and collectively as a healthcare system. The acronym stands for: Clinical, Administrative, Advocacy, Research, and Education. Let’s talk about each of them individually.”

Slide 20 - We CAARE: Clinical

“So, we can start with changes to our clinical practice and recommendations that are healthy for our patients, but also healthy for our planet which we all depend on. Many of these things have co-benefits, meaning not only benefitting our planet but also levels of societal health. Let’s list a few:

- We can recommend our patients be more active in their commute by walking or biking, and recommend eating a more plant-based diet.
- We can reduce hospital waste and the utilization of healthcare resources. We can use meds and anesthetics with less carbon footprint. We can make hospital wide changes toward climate goals. And we can protect our vulnerable patients as they are most at risk of climate change effects.
- Patient communication is particularly important, including educating our patients about the many health impacts expected from climate change. As mental health professionals, this also encompasses helping our patients navigate the very real and overwhelming world stressor of climate change, helping them develop healthy but realistic coping skills and perspectives.
- Below are a few of the resources to help in that transition and the next slide lists some resources to distribute to patients.

Slide 21 - Patient Materials

“These handouts and interactive modules are not only great for patients but can also be good brief reviews to educate ourselves about all the health effects of climate change.”

Slide 22 - How Does Healthcare Contribute?

“This is a good point to talk about how healthcare contributes to the climate crisis. The healthcare industry contributes almost 10% of U.S. greenhouse gas emissions (Eckelman & Sherman 2016). Some estimates put the US healthcare sector as the 7th largest emitter in the world if it were considered a country (<http://www.globalcarbonatlas.org/en/CO2-emissions>).

You will see a theme around supplies and supply chain fulfillment. It is estimated that 60-80% of healthcare emissions comes from supply chain fulfillment, so if we purchase more locally, reduce and reuse our supplies, use more renewable forms of energy and transportation, and create more virtual opportunities within healthcare then we can make major changes!”

Slide 23 - We CAARE: Administrative

“When I think of administrative action for climate and health, I think of how our health systems can interact with change in two ways. First, we can look externally with a public health lens, studying and advocating for interventions that alleviate the worst effects of the climate crisis.

Then, we can look internally at how our own health systems can improve their impact on the environment by cutting our waste and emissions. This can take many forms, including things like reimbursement metrics which account for sustainability benchmarks, and hospital commission standards which enforce sustainability and system resilience to climate change-related disasters.”

Slide 24 - We CAARE: Advocacy

“Polling consistently finds health professionals to be among the most trusted voices in society year after year. Part of what contributes to health professionals’ trust with the public is our role in communicating about important health issues. We can advocate for public health policy either collectively in organizations, or making direct appeals to the public through patient encounters or op-eds.

We can advocate and shape our institutions to contribute less to climate change and be more resilient to its effects. And we should advocate for our particularly vulnerable communities, generally through their partnership and guidance, to assist in preparing them for the effects of climate change.”

Slide 25 - We CAARE: Research

“Our research practices should also account for climate change. This can be done by contributing to gaps in knowledge about how climate change impacts health but also about the benefits of interventions. It can also be done by improving our research practices footprint and ensuring that our research practices are equitable by looking at impacts to communities that are marginalized racially and economically, in addition to metropolitan areas.”

Slide 26 - We CAARE: Education

“And finally education; we need to educate ourselves so we can educate others. There are some great courses to take online on our time which are really all encompassing of the health impacts of climate change. But ultimately, we need to make it more integral within health professional education. There are a few resources and organizations leading the charge on this, including the Global Consortium on Climate and Health Education (GCCHE), as well as The Medical Society Consortium on Climate and Health (MSCCH). Both of which have PowerPoint slide content to help educate on specific topics, recorded lectures, and GCCHE even has core competencies for health professional students.”

Slide 27 - Resources for Action: “Here are a bunch of resources you can peruse to educate yourself and help make the healthcare system more sustainable.”

Slide 28 - Conclusions: Read through the conclusions overview.

Slide 29/30 - References: To mention at the end. These references are listed in the slide deck and correspond to the various literature listed in the above presenter notes.

**Comments:**

This content can be emotionally distressing for students and should inform the facilitator’s administration of this educational session. However, just as we would counsel our patients not to avoid uncomfortable topics, we should not avoid uncomfortable topics with our students. This educational session was designed to end with solution-oriented content that can provide agency and coping skills for students. Providing solutions as part of ‘meaning-focused coping’ can help improve positive affect despite potentially emotionally unmooring content (Ojala 2012).

**References**:

<https://unfccc.int/files/adaptation/groups_committees/loss_and_damage_executive_committee/application/pdf/online_guide_on_loss_and_damage-dec_2017.pdf>. Retrieved June 7th, 2021.

Hoerling, M., Eischeid, J., Perlwitz, J., Quan, X., Zhang, T., & Pegion, P. (2012). On the increased frequency of Mediterranean drought. *Journal of climate*, *25*(6), 2146-2161.

Parida, Y., Dash, D. P., Bhardwaj, P., & Chowdhury, J. R. (2018). Effects of drought and flood on farmer suicides in Indian states: an empirical analysis. *Economics of disasters and climate change*, *2*(2), 159-180.

Hanigan, I. C., Butler, C. D., Kokic, P. N., & Hutchinson, M. F. (2012). Suicide and drought in new South Wales, Australia, 1970–2007. *Proceedings of the National Academy of Sciences*, *109*(35), 13950-13955.\

Gleick, P. H. (2014). Water, drought, climate change, and conflict in Syria. *Weather, Climate, and Society*, *6*(3), 331-340.

Smiatek, G., Kaspar, S., & Kunstmann, H. (2013). Hydrological climate change impact analysis for the Figeh Spring near Damascus, Syria. *Journal of Hydrometeorology*, *14*(2), 577-593.

Bozkurt, D., & Sen, O. L. (2013). Climate change impacts in the Euphrates–Tigris Basin based on different model and scenario simulations. *Journal of hydrology*, *480*, 149-161.

Hsiang, S.M., Burke, M., Miguel, E. (2013). Quantifying the influence of climate on human conflict. *Science*, 341, 6151.

Hauer, M. E., Evans, J. M., & Mishra, D. R. (2016). Millions projected to be at risk from sea-level rise in the continental United States. *Nature Climate Change*, *6*(7), 691-695.

Air Pollution - The Silent Killer. World Health Organization. Retrieved from https://www.who.int/multi-media/details/air-pollution-silent-killer. Published November 17, 2021. Accessed November 6, 2023.

Marino, E., & Lazrus, H. (2015). Migration or forced displacement?: the complex choices of climate change and disaster migrants in Shishmaref, Alaska and Nanumea, Tuvalu. *Human Organization*, (2015), 341-350.

Boyd, R. (2019, September 23). The People of the Isle de Jean Charles Are Louisiana's First Climate Refugees-but They Won't Be the Last. Retrieved March 14, 2020, from https://www.nrdc.org/stories/people-isle-jean-charles-are-louisianas-first-climate-refugees-they-wont-be-last

Khan, A. E., Xun, W. W., Ahsan, H., & Vineis, P. (2011). Climate change, sea-level rise, & health impacts in Bangladesh. *Environment: Science and Policy for Sustainable Development*, *53*(5), 18-33.

Few, R., Ahern, M., Matthies, F., & Kovats, S. (2004). Floods, health and climate change: a strategic review.

[The Air Quality Life Index (AQLI). (n.d.). Retrieved February 23, 2020, from <https://aqli.epic.uchicago.edu/pollution-facts/>]

Criteria Air Pollutants. (2018, March 8). Retrieved March 15, 2020, from https://www.epa.gov/criteria-air-pollutants

Environmental Fact Sheet. (2019). Retrieved March 15, 2020, from <https://www.des.nh.gov/organization/commissioner/pip/factsheets/ard/documents/ard-41.pdf>

<https://www.epa.gov/mercury/basic-information-about-mercury>. Retrieved June 6th, 2021.

Buoli, M., Grassi, S., Caldiroli, A., Carnevali, G. S., Mucci, F., Iodice, S., ... & Bollati, V. (2018). Is there a link between air pollution and mental disorders?. *Environment international*, *118*, 154-168.

Calderón-Garcidueñas, L., Calderón-Garcidueñas, A., Torres-Jardón, R., Avila-Ramírez, J., Kulesza, R. J., & Angiulli, A. D. (2015). Air pollution and your brain: what do you need to know right now. *Primary health care research & development*, *16*(4), 329-345.

Buoli, M., Serati, M., Caldiroli, A., Cremaschi, L., & Carlo Altamura, A. (2017). Neurodevelopmental versus neurodegenerative model of schizophrenia and bipolar disorder: comparison with physiological brain development and aging. *Psychiatria Danubina*, *29*(1), 24-27.

Hahad, O., Lelieveld, J., Birklein, F., Lieb, K., Daiber, A., & Münzel, T. (2020). Ambient air pollution increases the risk of cerebrovascular and neuropsychiatric disorders through induction of inflammation and oxidative stress. *International journal of molecular sciences*, *21*(12), 4306.

Abatzoglou, J. T., & Williams, A. P. (2016). Impact of anthropogenic climate change on wildfire across western US forests. *Proceedings of the National Academy of Sciences*, *113*(42), 11770-11775.

Graumann, A., Houston, T. G., Lawrimore, J. H., Levinson, D. H., Lott, N., McCown, S., ... & Wuertz, D. B. (2006). Hurricane Katrina: A climatological perspective: Preliminary report.

Calderon-Abbo, J. (2008). The long road home: Rebuilding public inpatient psychiatric services in post-Katrina New Orleans. *Psychiatric services*, *59*(3), 304-309.

Voelker, R. (2006). Post-Katrina mental health needs prompt group to compile disaster medicine guide. *Jama*, *295*(3), 259-260.

Kessler, R. C., Galea, S., Jones, R. T., & Parker, H. A. (2006). Mental illness and suicidality after Hurricane Katrina. *Bulletin of the World Health Organization*, *84*, 930-939.

Rudowitz, R., Rowland, D., & Shartzer, A. (2006). Health Care In New Orleans Before And After Hurricane Katrina: The storm of 2005 exposed problems that had existed for years and made solutions more complex and difficult to obtain. *Health Affairs*, *25*(Suppl1), W393-W406.

Manning, C., & Clayton, S. (2018). Threats to mental health and wellbeing associated with climate change. In *Psychology and climate change* (pp. 217-244). Academic Press.

https://www.climate.gov/news-features/understanding-climate/climate-change-global-temperature

Thompson, R., Hornigold, R., Page, L., & Waite, T. (2018). Associations between high ambient temperatures and heat waves with mental health outcomes: a systematic review. *Public health*, *161*, 171-191.

Basu, R., Gavin, L., Pearson, D., Ebisu, K., & Malig, B. (2018). Examining the association between apparent temperature and mental health-related emergency room visits in California. *American journal of epidemiology*, *187*(4), 726-735.

Burke, M., González, F., Baylis, P., Heft-Neal, S., Baysan, C., Basu, S., & Hsiang, S. (2018). Higher temperatures increase suicide rates in the United States and Mexico. *Nature climate change*, *8*(8), 723-729.

Dumont, Caroline; Haase, Elizabeth, Dolber, Trygve M; Lewis, Janet; Coverdale, John. Climate Change and Risk of Completed Suicide, The Journal of Nervous and Mental Disease: March 20, 2020 - Volume Publish Ahead of Print - Issue - doi: 10.1097/NMD.0000000000001162 Anderson, C. A. (2001). Heat and violence. *Current directions in psychological science*, *10*(1), 33-38.

Burke, M., González, F., Baylis, P. *et al.* (2018). Higher temperatures increase suicide rates in the United States and Mexico. *Nature Clim Change* 8, 723–729. doi: 10.1038/s41558-018-0222-x

Albrecht, G., Sartore, G. M., Connor, L., Higginbotham, N., Freeman, S., Kelly, B., ... & Pollard, G. (2007). Solastalgia: the distress caused by environmental change. *Australasian psychiatry*, *15*(sup1), S95-S98.

Higginbotham, N., Connor, L., Albrecht, G., Freeman, S., & Agho, K. (2006). Validation of an environmental distress scale. *EcoHealth*, *3*(4), 245-254.

Sartore, G. M., Kelly, B., Stain, H., Albrecht, G., & Higginbotham, N. (2008). Control, uncertainty, and expectations for the future: a qualitative study of the impact of drought on a rural Australian community.

Hendryx, M., & Innes-Wimsatt, K. A. (2013). Increased risk of depression for people living in coal mining areas of central Appalachia. *Ecopsychology*, *5*(3), 179-187.

Hsiang, S.M., Burke, M., Miguel, E. (2013). Quantifying the influence of climate on human conflict. *Science*, 341, 6151.

Gleick. (2014). Water, drought, climate change, and conflict in Syria. *Weather, Climate, and Society*, 6(3): 331-340.

Armstrong, A. K., Krasny, M. E., & Schuldt, J. P. (2018). *Communicating climate change: a guide for educators*. Comstock Publishing Associates.

Burke, S. E., Sanson, A. V., & Van Hoorn, J. (2018). The psychological effects of climate change on children. *Current psychiatry reports*, *20*(5), 1-8.

Clayton, S., & Karazsia, B. T. (2020). Development and validation of a measure of climate change anxiety. *Journal of Environmental Psychology*, *69*, 101434.

Crowley, R. A. (2016). Climate change and health: a position paper of the American College of Physicians. *Annals of internal medicine*, *164*(9), 608-610.

Doppelt, 2018. *Introduction To The Transformational Resilience Program* [PowerPoint slides]. http://www.theresourceinnovationgroup.org/

Herring, A. (2012, March 22). *3Qs: What is 'global weirding'?* Phys.org. https://phys.org/news/2012-03-3qs-global-weirding.html.

Hrabok, M., Delorme, A., & Agyapong, V. I. (2020). Threats to mental health and well-being associated with climate change. *Journal of Anxiety Disorders*, *76*, 102295.

Klonek, F. E., Güntner, A. V., Lehmann-Willenbrock, N., & Kauffeld, S. (2015). Using Motivational Interviewing to reduce threats in conversations about environmental behavior. *Frontiers in psychology*, *6*, 1015.

Lewis, J. L., Haase, E., & Trope, A. (2020). Climate Dialectics in Psychotherapy: Holding Open the Space Between Abyss and Advance. *Psychodynamic psychiatry*, *48*(3), 271-294.

Maclean, J. C., Popovici, I., & French, M. T. (2016). Are natural disasters in early childhood associated with mental health and substance use disorders as an adult?. *Social Science & Medicine*, *151*, 78-91.

Magalhaes, S. S., Malloy-Diniz, L. F., Rosa, D. V., Alvim-Soares, A., de Miranda, D. M., & Romano-Silva, M. A. (2020). Extreme Climate Related Disasters: two-time points evaluation of the impact in children and youth mental health. *medRxiv*.

Mah, A. Y., Chapman, D. A., Markowitz, E. M., & Lickel, B. (2020). Coping with climate change: Three insights for research, intervention, and communication to promote adaptive coping to climate change. *Journal of Anxiety Disorders*, *75*, 102282.

Mort, M., Walker, M., Williams, A. L., & Bingley, A. (2018). Displacement: critical insights from flood-affected children. *Health & place*, *52*, 148-154.

Niebert, K., & Gropengießer, H. (2014). Understanding the greenhouse effect by embodiment–analysing and using students' and scientists' conceptual resources. *International Journal of Science Education*, *36*(2), 277-303.

Ojala, M. (2012). How do children cope with global climate change? Coping strategies, engagement, and well-being. *Journal of Environmental Psychology*, *32*(3), 225-233.

Ojala, M. (2013). Coping with climate change among adolescents: Implications for subjective well-being and environmental engagement. *Sustainability*, *5*(5), 2191-2209.

Simmons, D. (2020, October 23). *How to talk with kids about climate change " Yale Climate Connections*. Yale Climate Connections. https://yaleclimateconnections.org/2020/08/how-to-talk-with-kids-about-climate-change/.

Semenza, J. C., Ploubidis, G. B., & George, L. A. (2011). Climate change and climate variability: personal motivation for adaptation and mitigation. *Environmental Health*, *10*(1), 1-12.

Stanley, S. K., Hogg, T. L., Leviston, Z., & Walker, I. (2021). From anger to action: Differential impacts of eco-anxiety, eco-depression, and eco-anger on climate action and wellbeing. *The Journal of Climate Change and Health*, *1*, 100003.

Streiffer, R. H., & Nagle, J. P. (2000). Patient education in our offices. *Journal of Family Practice*, *49*(4), 327-327.

Van Bavel, J. J., Baicker, K., Boggio, P. S., Capraro, V., Cichocka, A., Cikara, M., ... & Willer, R. (2020). Using social and behavioural science to support COVID-19 pandemic response. *Nature Human Behaviour*, 1-12.

Weems, C. F., Russell, J. D., Neill, E. L., Berman, S. L., & Scott, B. G. (2016). Existential anxiety among adolescents exposed to disaster: Linkages among level of exposure, PTSD, and depression symptoms. *Journal of traumatic stress*, *29*(5), 466-473.

Ojala, M. (2012). How do children cope with global climate change? Coping strategies, engagement, and well-being. *Journal of Environmental Psychology*, 32(3), 225-233.
