# Supplementary material for: Climate Change and Mental Health: An Interactive Educational Session
Source: MedEdPORTAL. 2024 Apr 19;20:11418. doi: 10.15766/mep_2374-8265.11418 (PMC11026302; doi:10.15766/mep_2374-8265.11418)
Supplement: Supplementary file 1 — Session Presentation.pptxFacilitator Guide.docxPostsession Resources for Students.docxPre- and Postsession Survey.docx [file mep_2374-8265.11418-s001.zip › _Educational Summary Report_11418.pdf]

MedEdPORTAL® | The AAMC Journal of Teaching and Learning Resources

Original Publication Open Access

Climate Change and Mental Health: An Interactive Educational Session

Andrea Costin, MPH, Daniel Fisher, MD, Bethany Harper, MD, Ramzi W. Nahhas, PhD, John Sullenbarger, MD*

*Corresponding author: john.sullenbarger@wright.edu

Abstract

Introduction: Climate change is the single biggest health threat facing humanity, with direct and indirect impacts on mental health, yet health impacts of climate change remain notably absent from most medical school curricula. We describe a timely interactive educational session on climate change and mental health that was implemented and studied on a medical student clinical psychiatry rotation. **Methods:** We developed a 1-hour introductory session on the mental health impacts of climate change and potential solutions. The session was delivered to third-year medical students on their 4-week clinical psychiatry rotation and included pre- and postsession survey questions assessing their knowledge, comfort, and readiness regarding the topic. **Results:** Seventy students participated in the session, with 49 students completing the pre- and postsession surveys, giving a response rate of 70%. The average score for the four Likert-scale questions on the survey increased from 2.7 presession to 3.9 postsession on a 5-point scale (1 = *strongly disagree,* 5 = *strongly agree*). All questions displayed statistically significant improvement. Qualitative analysis identified knowledge gained about the mental health impacts of climate change as the most important aspect of the session to students. **Discussion:** The introductory session effectively filled an urgent need in medical education curricula regarding climate change's effects on human health. Overall, distribution of and improvement upon this timely teaching content can serve a valuable role in medical student education as the effects of climate change, particularly on mental health, continue to progress throughout the century.

Keywords
Disasters, Extreme Heat, Global Warming, Neurology, Neuroscience, Population Health, Psychiatry, Psychology & Behavioral Science, Well-Being/Mental Health, Climate Change

Educational Objectives

By the end of this activity, learners will be able to:

1. List psychiatric conditions and other mental health impacts that emerge from and/or are affected by the climate crisis.
2. Describe methods of climate communication in the clinical setting.
3. Compare roles that health professionals should perform in facilitating resilience and pro-environmental behaviors.
4. Describe available resources to facilitate such public health and mental health activities.

Introduction

Climate change, driven primarily by greenhouse gas emissions from human combustion of fossil fuels, is a known global disruptor of planetary systems. This has led to rising global temperatures and slow-moving disasters like drought and sea level rise, as well as acute disasters like wildfires, heat waves, and extreme weather. As climate change has become increasingly accepted, research on how climate and health are intertwined has increased elevenfold between 2007 and 2020,[1] revealing numerous impacts on human health. Consequently, climate change has been labeled "the single biggest health threat facing humanity" by the World Health Organization.[2]

Mental health has also been affected by climate change, specifically through direct, indirect, and psychological impacts.[3] Direct impacts on mental health include "the neurobiologic and psychiatric symptom responses to exposure to climate drivers," while among the indirect impacts are "pervasive and long-lasting mental health consequences through significant losses" from climate change.[4] Extreme heat provides many examples of direct and indirect impacts. It causes direct mental health impacts due to higher associated rates of suicide and violence,[5-7] heat-related deaths for those with mental illness and on psychotropics,[8] and reduced working memory, reaction times, and attention.[9]

Citation:
Costin A, Fisher D, Harper B, Nahhas RW, Sullenbarger J. Climate change and mental health: an interactive educational session. *MedEdPORTAL*. 2024;20:11418.
https://doi.org/10.15766/mep_2374-8265.11418

Copyright © 2024 Costin et al. This is an open-access publication distributed under the terms of the Creative Commons Attribution license.

Meanwhile, extreme heat will likely render many places difficult to inhabit, thus causing indirect mental health impacts from migration, forced time indoors, and alterations of surrounding landscapes. In contrast, psychological impacts encompass all the various emotional and cognitive responses to climate change. These include anxiety, grief, denial, hopelessness, and more; these responses are collectively known as climate distress. A study of 10,000 youth in 10 countries revealed that 84% had at least moderate anxiety about climate change, while 45% experienced daily negative impacts in their lives from climate distress.[10]

As the medical community increasingly recognizes the rising health threat of climate change through epidemiologic studies and climate modeling, incorporating climate health education into medical school curricula has become increasingly necessary.[11] Many calls to increase climate health in medical school curricula have recently arisen and have expressed the need to prepare a psychiatric workforce for increasing demands from climate change,[12] though a study in 2020 found that only 15% of medical schools out of 2,817 in 112 countries included climate change and health in their curricula.[13] Reasons for this absence from curricula have been postulated to include medical professionals' own cognitive biases or climate-related anxiety, a lack of resources and local experts to facilitate learning, shrinking curriculum space for important topics like climate change and health impacts, and minimal evaluation data on various climate and mental health curricular outcomes.[3] It is timely to include climate change curricula during the clinical years of medical school due to how this global phenomenon engages critical thinking in students and requires patient empathy. An education session on the health impacts of climate change held within the psychiatry clerkship setting is novel to medical education, to our knowledge. It is important to have it in the clerkship due to the juxtaposition this topic has with direct patient experiences, as well as the breadth and critical thinking required to address the problem throughout medicine's practice, meaning that students should have early exposure to the topic prior to residency. In order to help address this absence, we developed a timely interactive introductory session on climate change and mental health for medical students on their psychiatry rotation, provided materials to assist educators in administering the content, and studied the effectiveness of the material at improving students' knowledge of climate change and mental health.

Methods

An introductory curriculum called Climate Change and Human Health was originally developed by the senior author, a steering committee member of the Climate Psychiatry Alliance (CPA),[14] with input from CPA collaborating providers. This material was updated and adapted to provide a more interactive learning experience and highlight the most impactful effects of climate change on mental health while generalizing actionable items for unspecialized learners early in their medical education. Emphasis was placed on describing the direct and indirect psychiatric impacts, as well as psychological effects, from climate change and its drivers (e.g., extreme weather events, wildfires, droughts, severe heat, etc.) before shifting to generalizable actions for all health care providers.

Implementation

We developed this interactive, educational, and introductory session for third-year medical students rotating on their 4-week required psychiatry clerkship at the Boonshoft School of Medicine at Wright State University. To maximize the session's impact, empower students, and allow the opportunity to apply the content in real time, the session was incorporated into required third-year clerkship didactics during a time when students would be interacting directly with patients. Students completed their introduction to psychiatry course at the end of their second year of medical school, which ensured that they would have a solid foundation of knowledge about psychiatric disorders.

Our 1-hour interactive session was given every 4 weeks from August 2021 to June 2022 for 12 sessions. There were no presession materials provided. We routinely administered the session in a synchronous virtual format during the third week of the psychiatry clerkship, though the timing of the session during the rotation, as well as the format (i.e., in person vs. asynchronous virtual), was sometimes altered to accommodate schedule availability. The session was provided in person and virtually via recording once each. The session was first administered as a lunch-hour session while students were at their clinical sites. However, to improve attendance and survey response rates, the session was later integrated into the scheduled clerkship rotation didactics.

Curricular Materials

After introducing the topic and stating the learning objectives, the session began with an interactive discussion with students regarding the conceptualization of climate change and gauging their knowledge level on climate change effects on human health, particularly mental health. Appendix A is a PowerPoint for instructing medical students, and Appendix B is a facilitator guide to help the facilitator talk through the presentation. The course content was broken into three distinct sections: (1) an overview of mental health impacts from climate change and its drivers

Copyright © 2024 Costin et al. This is an open-access publication distributed under the terms of the Creative Commons Attribution license.

(i.e., drought, sea level rise, air pollution, wildfires, severe storms, and extreme heat), (2) psychological effects from and responses to climate change, and (3) actionable items enabling health care providers to address climate change along with a list of currently available resources. Actionable items were broken into five focus areas summated by the acronym We CAARE devised within the CPA: clinical, administrative, advocacy, research, and education. These actionable items are described in further detail in Appendix C, which provides resources for students' at-home review after the session.

Evaluation of Content

The four Educational Objectives of this project were designed to build on the students' current knowledge base of psychiatric disorders through large-group discussion, and survey questions were designed to assess the efficacy of the learning session at meeting these aims. The pre- and postsession survey used is provided in Appendix D.

Students invited to attend the session were sent pre- and postsession surveys via email. They were given 1-2 weeks to complete the presession survey and had 5 minutes at the end of the session to complete the postsession survey. The survey consisted of the same four questions, based on the above aims, asked before and after the course. Each question was scored on a 5-point Likert scale (1 = *strongly disagree,* 5 = *strongly agree*). The postsession survey additionally asked for short answers on what content students found to be the most important learned during the course as well as areas for improvement. Quantitative statistical analysis was completed within the Department of Psychiatry at Wright State University, with a primary analysis comparing average scores for each question and an overall score averaging each question together. Qualitative analysis was performed on the postsession survey short-answer responses through thematic identification.

Results

Seventy clerkship students participated in the session. Among them, 49 completed matched pre- and postevaluations, for a response rate of 70%. The four Likert-scale questions were scored on a 5-point scale (1 = *strongly disagree,* 5 = *strongly agree*). The results demonstrated that students agreed significantly more with the four items postsession than presession. This was true overall, with the average total score for the four questions increasing from 2.7 to 3.9 ($SD = 1.5$, $p <$ .001; 95% confidence interval [CI], 0.9-1.5) as well as for each of the four items individually (Table). The largest increase came from question 4, "I am aware of available resources on climate change and human health to support my actions as a health professional," with an increase from 2.2 to 4.0 ($p < .001$; CI, 1.4-2.0). The increase represents a transition from only three presession responses agreeing or strongly agreeing with the statement to 38 postsession responses agreeing or strongly agreeing with the statement. Question 2, "I feel comfortable communicating to patients and peers about climate change and human health," displayed the lowest postevaluation score, with a 3.7. On question 2, the number of students disagreeing or strongly disagreeing decreased from 28 to five following the session (Figure).

Table. Student Changes in Average Scores

	M (*SD*)	
**Question**[a]	**Presession**[b]	**Postsession**[b]
Total average score	2.7 (0.7)	3.9 (0.7)
Q1: Knowledgeable	2.9 (1.1)	4.1 (0.7)
Q2: Comfortable	2.6 (1.0)	3.7 (0.9)
Q3: Prepared	3.2 (1.0)	3.9 (0.9)
Q4: Aware	2.2 (0.8)	4.0 (0.8)

[a]Rated on a 5-point Likert scale (1 = *strongly disagree,* 5 = *strongly agree*).
[b]*N* = 49 (50%).

Students responded to two open-ended questions to identify the most impactful content from the course and provide suggestions for improvement. Thematic analysis of each question revealed that students identified their increase in knowledge about the mental health impacts of climate change as being the most important thing learned during the session (25 of 49 respondents), with many having listed meaningful facts they learned during the session. Other areas of the session students found particularly impactful included learning about resources (four of 49), feeling more prepared to help address climate change (five of 49), how health care systems could be improved (five of 49), and how health care professionals could include this

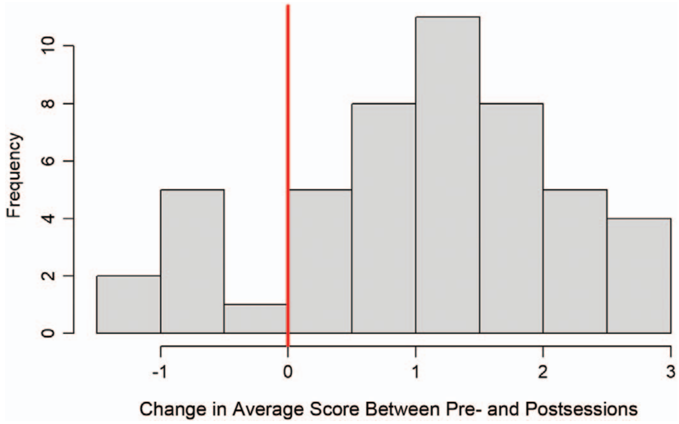

Figure. Change in average score.

Copyright © 2024 Costin et al. This is an open-access publication distributed under the terms of the Creative Commons Attribution license.

content in their clinical practice (four of 49). Students mainly offered isolated comments or provided no suggestions for improvement (37 of 49). However, some themes identified included students' desires to increase the interactive component of the session (six of 49), make the content more clinically oriented (two of 10), and increase the amount of resources (three of 49) that they could review.

Discussion

Climate change has increasingly been recognized as an urgent threat to human health, including mental health, with current health impacts progressively increasing in proportion to greater exposure to climate drivers, such as severe storms, heat waves, and more. Despite this identified threat to human health, there have been few reported efforts to integrate the mental health impacts of climate change into medical school and residency curricula. Our interactive educational session was developed to fill this urgent gap and, to our knowledge, is one of the first efforts to implement and study the efficacy of a climate change curriculum in a medical school psychiatry rotation.

In the learning session, we chose specific content to connect basic psychiatric knowledge with how climate change exacerbates mental health. We further included corresponding interventions to improve students' sense of empowerment. In addition to offering actionable steps for health care providers, we utilized intentional debriefing at the session's conclusion to counter internal climate distress that may have been provoked in students due to discussing a difficult topic. This sense of agency and direct confrontation of a distressing topic may improve students' ability to take action aligned with their values, similar to meaning-focused coping (where people draw on their beliefs, values, and existential goals to sustain well-being), which has been shown to improve positive affect.[15] While our session focused primarily on mental health impacts of climate change, students with varied specialty interests learned how to integrate the content into medical practice. This was an important approach since only approximately 7% of medical students chose to pursue a career in psychiatry in 2023.[16]

Survey results showed that our interactive educational session was effective at meeting its four Educational Objectives. Prior to the session, scores were low for each of the four survey questions corresponding with the session's four Educational Objectives, and there was a statistically significant increase when comparing postsession results to presession. These results, combined with the qualitative analysis results above, reflect the session's efficacy in bolstering students' familiarity with timely content in a psychiatry clerkship, a strength of the session. However, while these results show increases in immediate familiarity with the content and ability to take actionable steps, there are no data to illuminate students' application of the knowledge in clinical practice. One future area of interest would be a longitudinal study following recipients of this learning session and how they implement it in clinical practice.

Another area of improvement would involve students' qualitative feedback about desiring greater interactivity in the learning session. While attempts were made to increase learners' active engagement, the discussion was paired with didactic-style slides in order to provide adequate depth and breadth of knowledge in a short 1-hour learning session without adding outside preparatory materials. Future implementers of this session might consider editing the material to pair it with, or completely comprise, case-based learning in order to highlight the important content while increasing interactivity.

Additionally, the educational session was developed at a point when the length of the psychiatry clerkship decreased from 6 to 4 weeks and the time available for structured session content was limited, a common trend in medical school curricular schedules. We developed the session to include active learning discussion paired with informational slides, which allowed the topic to be formally integrated into the curriculum while minimizing the burden of preparatory material. The session was first offered virtually during the lunch hour from noon to 1:00 p.m. as a voluntary activity. Due to low attendance, however, it was integrated into the weekly required clerkship didactics. This change resulted in core content being consolidated and restructured to meet the learning objectives of the rotation. This change to core content may not be feasible for or deemed desirable by some directors of medical student education in psychiatry. Therefore, to integrate essential and timely material on climate change and mental health, it may prove beneficial to develop asynchronous learning materials for utilization outside structured didactic time and to integrate climate change's health impacts within all core content, in both the psychiatry rotation and the greater medical school curriculum.

This interactive educational session effectively served as an introduction within our medical school's curriculum to how climate change is affecting mental health, as well as to the corresponding actions for health care providers. We found the session efficacious in improving students' familiarity with the topic

Copyright © 2024 Costin et al. This is an open-access publication distributed under the terms of the Creative Commons Attribution license.

overall. Many medical schools may have a dearth of teachers who feel comfortable devising material and instructing on this content; thus, the accompanying PowerPoint slides, facilitator guide, and student resources can assist with broader instruction about this important topic. While psychiatric educators have a unique advantage in addressing barriers such as denial and behavioral passivity, widespread inclusion of this content in medical professional education will be vital to ensure both the health care sector's readiness to adapt to climate change's health impacts and innovation to mitigate the worst of the effects.

While our project provides insight into integrating content on the mental health impacts of climate change into the psychiatry clerkship, it is valuable to acknowledge some limitations. We selected a short Likert-based pre- and postsurvey format using objective and subjective prompts to evaluate the effectiveness of the session. This reliance on self-assessment could have led to overestimation of the session's academic impact. An objective knowledge-based assessment could have provided higher-level data to illustrate successfully achieving the Educational Objectives. With a 70% response rate, there remains concern about nonresponder bias and the potential change to the outcome measures if the missing 30% of participants had completed the postsession survey. Our intervention and postevaluation offer a time-limited view of the intervention's impact. There are no data on this onetime educational session's long-term retention or impact.

Appendices

A. Session Presentation.pptx

B. Facilitator Guide.docx

C. Postsession Resources for Students.docx

D. Pre- and Postsession Survey.docx

All appendices are peer reviewed as integral parts of the Original Publication.

Andrea Costin, MPH: Fourth-Year Medical Student, Wright State University Boonshoft School of Medicine; ORCID: https://orcid.org/0009-0006-7869-8738

Daniel Fisher, MD: Third-Year Resident, Department of Psychiatry, Wright State University

Bethany Harper, MD: Director of Medical Student Education in Psychiatry and Associate Professor, Department of Psychiatry, Wright State University

Ramzi W. Nahhas, PhD: Professor, Department of Population and Public Health Sciences and Department of Psychiatry, Wright State University

John Sullenbarger, MD: Assistant Residency Training Program Director and Assistant Professor, Department of Psychiatry, Wright State University; https://orcid.org/0009-0000-7991-8700

Acknowledgments

The authors greatly appreciate and acknowledge the Climate Psychiatry Alliance for their contribution to this work.

Disclosures

None to report.

Funding/Support

None to report.

Prior Presentations

Costin A, Fisher D, Sullenbarger J, Harper B. Climate change and mental health: filling an urgent curriculum gap. Oral presentation presented at: Association for Directors of Medical Student Education in Psychiatry Annual Meeting; June 24, 2023; San Diego, CA.

Fisher D, Costin A, Sullenbarger J, Harper B. Climate change and mental health: filling an urgent curriculum gap. Poster presented at: Association for Directors of Medical Student Education in Psychiatry Annual Meeting; June 23, 2023; San Diego, CA.

Ethical Approval

The Wright State University Institutional Review Board deemed further review of this project not necessary.

References

1. Romanello M, McGushin A, Di Napoli C, et al. The 2021 report of the *Lancet* Countdown on health and climate change: code red for a healthy future. *Lancet.* 2021;398(10311):1619-1662. https://doi.org/10.1016/S0140-6736(21)01787-6
2. Climate change and health. World Health Organization. 2021. Accessed January 6, 2022. https://www.who.int/news-room/fact-sheets/detail/climate-change-and-health
3. Hayes K, Blashki G, Wiseman J, Burke S, Reifels L. Climate change and mental health: risks, impacts and priority actions. *Int J Ment Health Syst.* 2018;12:28. https://doi.org/10.1186/s13033-018-0210-6
4. Sullenbarger J, Schutzenhofer E, Haase E. Climate change: implications for community mental health. In: Sowers WE, McQuistion HL, Ranz JM, Feldman JF, Runnels RS, eds. *Textbook of Community Psychiatry*. 2nd ed. Springer; 2022:427-442.
5. Burke M, González F, Baylis P, et al. Higher temperatures increase suicide rates in the United States and Mexico. *Nat Clim Chang.* 2018;8:723-279. https://doi.org/10.1038/s41558-018-0222-x

Copyright © 2024 Costin et al. This is an open-access publication distributed under the terms of the Creative Commons Attribution license.

6. Dumont C, Haase E, Dolber T, Lewis J, Coverdale J. Climate change and risk of completed suicide. *J Nerv Ment Dis*. 2020;208(7):559-565. https://doi.org/10.1097/NMD.0000000000001162
7. Hsiang SM, Burke M, Miguel E. Quantifying the influence of climate on human conflict. *Science*. 2013;341(6151):1235367. https://doi.org/10.1126/science.1235367
8. Bouchama A, Dehbi M, Mohamed G, Matthies F, Shoukri M, Menne B. Prognostic factors in heat wave-related deaths: a meta-analysis. *Arch Intern Med*. 2007;167(20):2170-2176. https://doi.org/10.1001/archinte.167.20.ira70009
9. Schlader ZJ, Gagnon D, Adams A, Rivas E, Cullum CM, Crandall CG. Cognitive and perceptual responses during passive heat stress in younger and older adults. *Am J Physiol Regul Integr Comp Physiol*. 2015;308(10):R847-R854. https://doi.org/10.1152/ajpregu.00010.2015
10. Hickman C, Marks E, Pihkala P, et al. Climate anxiety in children and young people and their beliefs about government responses to climate change: a global survey. *Lancet Planet Health.* 2021;5(12):e863-e873. https://doi.org/10.1016/S2542-5196(21)00278-3
11. Seritan AL, Coverdale J, Brenner AM. Climate change and mental health curricula: addressing barriers to teaching. *Acad Psychiatry.* 2022;46(5):551-555. https://doi.org/10.1007/s40596-022-01625-0
12. Hwong AR, Kuhl EA, Compton WM, et al. Climate change and mental health: implications for the psychiatric workforce. *Psychiatr Serv*. 2022;73(5):592-595. https://doi.org/10.1176/appi.ps.202100227
13. El Omrani O, Dafallah A, Castillo PB, et al. Envisioning planetary health in every medical curriculum: an international medical student organization's perspective. *Med Teach*. 2020;42(10):1107-1111. https://doi.org/10.1080/0142159X.2020.1796949
14. Climate Psychiatry Alliance. Accessed March 26, 2024. https://www.climatepsychiatry.org/
15. Ojala M. How do children cope with global climate change? Coping strategies, engagement, and well-being. *J Environ Psychol*. 2012;32(3):225-233. https://doi.org/10.1016/j.jenvp.2012.02.004
16. National Resident Matching Program. *Results and Data: 2023 Main Residency Matches. National Resident Matching Program*; 2023. Accessed March 26, 2024. https://www.nrmp.org/wp-content/uploads/2023/05/2023-Main-Match-Results-and-Data-Book-FINAL.pdf

Received: November 8, 2023
Accepted: March 14, 2024
Published: April 19, 2024

Copyright © 2024 Costin et al. This is an open-access publication distributed under the terms of the Creative Commons Attribution license.
